# Energy homeostasis is a conserved process: Evidence from *Paracoccus denitrificans*’ response to acute changes in energy demand

**DOI:** 10.1371/journal.pone.0259636

**Published:** 2021-11-08

**Authors:** Raul Covian, Lanelle Edwards, Yi He, Geumsoo Kim, Carly Houghton, Rodney L. Levine, Robert S. Balaban

**Affiliations:** 1 Laboratory of Cardiac Energetics, National Heart, Lung, and Blood Institute, National Institutes of Health, Bethesda, Maryland, United States of America; 2 Fermentation Facility, National Heart, Lung, and Blood Institute, National Institutes of Health, Bethesda, Maryland, United States of America; 3 Laboratory of Biochemistry, National Heart, Lung, and Blood Institute, National Institutes of Health, Bethesda, Maryland, United States of America; National Research Council, ITALY

## Abstract

*Paracoccus denitrificans* is a model organism for the study of oxidative phosphorylation. We demonstrate a very high respiratory capacity compared to mitochondria when normalizing to cytochrome *aa*_3_ content even in the absence of alternative terminal oxidases. To gain insight into conserved mechanisms of energy homeostasis, we characterized the metabolic response to K^+^ reintroduction. A rapid 3-4-fold increase in respiration occurred before substantial cellular K^+^ accumulation followed by a sustained increase of up to 6-fold that persisted after net K^+^ uptake stopped. Proton motive force (Δ*p*) was slightly higher upon addition of K^+^ with ΔpH increasing and compensating for membrane potential (ΔΨ) depolarization. Blocking the F_0_F_1_-ATP synthase (Complex V) with venturicidin revealed that the initial K^+^-dependent respiratory activation was primarily due to K^+^ influx. However, the ability to sustain an increased respiration rate was partially dependent on Complex V activity. The 6-fold stimulation of respiration by K^+^ resulted in a small net reduction of most cytochromes, different from the pattern observed with chemical uncoupling and consistent with balanced input and utilization of reducing equivalents. Metabolomics showed increases in glycolytic and TCA cycle intermediates together with a decrease in basic amino acids, suggesting an increased nitrogen mobilization upon K^+^ replenishment. ATP and GTP concentrations increased after K^+^ addition, indicating a net increase in cellular potential energy. Thus, K^+^ stimulates energy generation and utilization resulting in an almost constant Δ*p* and increased high-energy phosphates during large acute and steady state changes in respiration. The specific energy consuming processes and signaling events associated with this simultaneous activation of work and metabolism in *P*. *denitrificans* remain unknown. Nevertheless, this homeostatic behavior is very similar to that observed in mitochondria in tissues when cellular energy requirements increase. We conclude that the regulation of energy generation and utilization to maintain homeostasis is conserved across the prokaryote/eukaryote boundary.

## Introduction

The purpose of this study was to evaluate the metabolic response to acute changes in energy demand in *Paracoccus denitrificans*, with special interest in oxidative phosphorylation. Based on the theory that mitochondria evolved from a symbiotic incorporation of bacteria into the eukaryotic cell [1,2], it is conceivable that the mechanism of respiratory control in mitochondria is conserved from bacteria, just as occurred with DNA and the protein synthesis machinery. Thus, the characterization of acute regulatory control of oxidative phosphorylation in bacteria may provide insight into the regulation of mitochondrial energy conversion in intact mammalian tissues, which remains poorly understood [3,4]. Based on isolated mitochondria studies, the classical model of regulating oxidative phosphorylation was proposed to be a simple feedback model of ADP and phosphate driving faster respiration rates when ATP hydrolysis increased [5–7]. However, one of the remarkable features of energy metabolism in striated muscle is the ability to maintain the concentration of high-energy phosphate metabolites during large changes in respiration and ATP hydrolysis rates, implying a precise matching of energy generation and utilization rates without compromising the free energy to perform work. This phenomenon was first described by A.V. Hill in 1950 [8] and was subsequently referred to as energy metabolism homeostasis [9,10]. Thus, the lack of change in cytosolic ATP, ADP, and phosphate with increases in respiration suggests other mechanisms regulate oxidative phosphorylation in intact systems. The signaling and control mechanisms involved in generating the metabolic homeostasis in mammalian tissues such as striated muscle remain unknown [11]. Since *P*. *denitrificans* is an excellent model for mitochondrial oxidative phosphorylation [12–14], we propose that an understanding of the acute modulation of energy metabolism in *P*. *denitrificans* will provide insights into the regulatory mechanisms of mitochondrial energy conversion.

The steady state metabolism of bacteria has been extensively studied and modeled in order to optimize the culturing of beneficial bacteria or prevent the growth of those that are pathogenic [15,16]. Work has primarily focused on the metabolic requirements for cell growth, and more recently, on the metabolic contributions to the mammalian gut [17], with few studies on the acute modulation of energy metabolism during rapid workload changes or the energy demands of maintaining cellular homeostasis. The most obvious acute work challenge in bacteria, not related to cell growth, involves maintaining ionic and osmotic gradients [18]. For example, microbial species living in the upper gastrointestinal system experience wide changes in osmolality associated with drinking fluids that require rapid adjustments to regenerate ion gradients. Bacteria living in the soil and other natural environments also experience large acute changes in ionic and osmotic conditions with different weather conditions that require rapid adjustments in energy metabolism.

Most cells and organelles, including bacteria and mitochondria, maintain a high internal K^+^ concentration. The reasons for the nearly ubiquitous high intracellular K^+^ are still not fully understood [19,20] although significant amounts of energy are expended maintaining the K^+^ gradient. The acute transition to a state of higher energy demand that we have studied in the present work is the response to K^+^ replenishment in *P*. *denitrificans* cells. Previous studies had established that reintroduction of K^+^ to these cells results in a rapid and sustained increase in respiratory activity [21,22]. The specific K^+^ uptake mechanisms in *P*. *denitrificans* have not been fully analyzed. This is due, at least in part, to the lack of genetic knockouts of the transporters, specific inhibitors, and proteomic analysis. Nevertheless, the genome of *P*. *denitrificans* [23] includes most of the known bacterial K^+^ transport systems, including the kdp ATPase [24], the Trk channel [25], and the pha K^+^/H^+^ exchanger [26]. The specific roles of these K^+^ transport systems in *P*. *denitrificans* under different experimental conditions have not been determined. It is not known what work function (*i*.*e*. ion transport, volume regulation, protein synthesis, or regulatory “futile” cycles) contribute to the observed increase in respiration [27]. The K^+^ dependent increase in respiration has been proposed to be associated with a higher NADH/NAD^+^ ratio and reduction of *c*-type cytochromes [21], suggesting an activation of intermediary metabolism by K^+^ either directly or indirectly [27]. Thus, the activation of energy production by K^+^ uptake is much more complex than the simple increase of ATPase activities or the dissipation of Δ*p* as K^+^ is brought into the cell.

Here, we quantitatively evaluate the metabolic effects of re-exposure to K^+^ in *P*. *denitrificans* strains grown in a defined aerobic medium with either malate or glucose as sole carbon and energy source. Intracellular K^+^ was efficiently depleted by exposing cells to a hyperosmotic shock of glycerol during harvesting followed by freezing. We have made proteomic analyses of protein expression to characterize the strains used, and determined the metabolomic profile resulting from the absence or presence of external K^+^. In addition to respiration, K^+^ uptake rates, and cytochrome redox state, Δ*p* was also dynamically measured by continuously monitoring ΔΨ and ΔpH using a novel pH-sensitive GFP expressing strain. Our results demonstrate that respiration in *P*. *denitrificans* can be instantly stimulated several-fold by re-exposure to K^+^ well before intracellular K^+^ concentration substantially increases. Additional activation of oxidative phosphorylation occurs as K^+^ uptake proceeds, resulting in a slight increase of Δ*p* and a significantly higher ATP content. The network responsible for these phenomena balances the rate of reducing equivalent delivery for Δ*p* generation with utilization by energy consuming processes. Minimal changes in the redox state of the cytochromes were observed, demonstrating that homeostatic control occurs over all steps of the oxidative phosphorylation cascade. We also inhibited protein synthesis and determined volume changes as well as heat generation to explore possible work functions (*i*.*e*., osmoregulation, synthesis of macromolecules or storage polymers) that could require the resulting increased energy provided by the acute metabolic activation induced by K^+^. Although the specific energy consuming processes activated by the acute replenishment of K^+^ in *P*. *denitrificans* could not be precisely identified, energy homeostasis was clearly evident in this bacterial system. These results suggest that the yet to be determined mechanisms that balance energy production and utilization in tissue mitochondria are also present in bacteria closely related to the first mitochondrial endosymbiont.

## Results

### Growth parameters of *P*. *denitrificans* strains with glucose or malate as carbon source

*P*. *denitrificans* has frequently been used as a model organism for the study of mitochondrial oxidative phosphorylation. One advantage of studying *P*. *denitrificans* is that it cannot ferment [28]; its energy production requires respiration under aerobic conditions. In addition to the *aa*_3_-type cytochrome *c* oxidase (Complex IV), this bacterium has two alternative terminal oxidases not present in mitochondria [29]. To allow a better comparison to mitochondrial oxidative phosphorylation, we generated strains lacking both alternative oxidase activities by deleting their respective oxygen binding subunits, encoded by the *cyoB* and *ccoN* genes. In addition to glucose, which is catabolized initially through glycolysis, malate was chosen as a carbon and energy source that bypasses glycolysis. Table 1 shows that the doubling time was similar (within 10%) between wild-type and the CyoB^-^/CcoN^-^ strain grown in the same substrate (glucose or malate). When cells were cultured with glucose, the doubling time was 20–40% longer than with malate in both strains. Oxygen consumption rates were ~30%higher when grown in malate than in glucose after normalization to the number of cells or to the content of Complex IV. The rate of CO_2_ generation in malate was ~2-fold higher than in glucose.

**Table 1:** Metabolic parameters of *P*. *denitrificans* strains grown in glucose or malate^a^.

Strain	Doubling time (min)	O_2_ consumption (mol O_2_/min/cell)	CO_2_ generation (mol CO_2_/min/cell)	RQ^b^	Cytochrome *aa*_3_ (mol/cell)	O_2_ consumption (mol O_2_/min/mol cyt *aa*_3_)
Wild-type Glucose (n = 5)	113 ± 0.7	3.3 ± 0.5 x 10^−17^	3.3 ± 0.04 x 10^−17^	1.0	1.93 ± 0.03 x 10^−20^	1700 ± 30
Wild-type Malate (n = 5)	81 ± 1	4.2 ±0.3 x 10^−17^	6.2 ± 0.2 x 10^−17^	1.5	1.89 ± 0.05 x 10^−20^	2230 ± 160
CyoB^-^/CcoN^-^Glucose (n = 6)	106 ± 0.6	3.3 ± 0.5 x 10^−17^	3.3 ± 0.5 x 10^−17^	1.0	2.30 ± 0.02 x 10^−20^	1450 ± 220
CyoB^-^/CcoN^-^Malate (n = 3)	89 ± 2	4.3 ± 0.2 x 10^−17^	7.0 ± 0.9 x 10^−17^	1.6	2.31 ± 0.02 x 10^−20^	1850 ± 70

Mean ± SE values are shown.

^a^Cells were harvested at an OD of 1.5 with the exception of 4 growths of CyoB^-^/CcoN^-^ glucose cells grown to 15 OD).

^b^Respiratory Quotient was calculated by dividing CO_2_ generation by O_2_ consumption close to the end of growth when O_2_ consumption values were more reliable.

### Relative quantitation of protein expression in wild-type and CyoB^-^/CcoN^-^ *P*. *denitrificans*

Proteomic analysis confirmed the absence of the *cbb*_3_ alternative oxidase by the markedly lowered expression of subunits II and III in the CyoB^-^/CcoN^-^ strain (S1 Table). The other two subunits associated with the *cbb*_3_ oxidase (including the deleted CcoN) were not detected. Of the four subunits comprising the alternative quinol oxidase to which the deleted CyoB^-^ protein belongs, only subunit II was detected. This subunit (encoded by the *cyoA* gene upstream of *cyoB*) was still expressed in the double mutant (~50% higher in glucose and ~70% lower in malate) relative to wild-type cells (S1 Table) but is not sufficient by itself to allow quinol oxidase activity if the other subunits are absent [29]. Soluble cytochrome *c*550 decreased by more than half in the absence of alternative oxidases. Only subunit II of Complex IV was significantly higher in the CyoB^-^/CcoN^-^ cells grown in glucose, although changes in the levels of the other three subunits of this complex were not statistically different, in agreement with the similar values obtained spectroscopically for cytochrome *aa*_3_ between strains (see Table 1). Only two of the fifteen detected Complex I subunits (J and K) were significantly lower in wild-type cells grown in glucose compared to malate (S1 Table). However, these highly hydrophobic subunits were not different in the CyoB^-^/CcoN^-^ cells when comparing between the two substrates. Of the detected K^+^ transport proteins, a small increase was significant only for the TrkH subunit in the CyoB^-^/CcoN^-^ grown on glucose. Growth in glucose or malate induced a very different expression of proteins such as carbohydrate and carboxylate transporters, as well as glycolytic and gluconeogenic enzymes, consistent with their known metabolic pathways (S2 Table). Besides subunits from the *cbb*_3_ alternative oxidase, a variety of proteins unrelated to oxidative phosphorylation showed variations in their content in CyoB^-^/CcoN^-^ cells grown in glucose (S3 Table) or malate (S4 Table). Changes in the expression of the >3000 proteins detected as a function of substrate and/or strain is provided as a single data file as Supporting information (S5 Table).

### Respiratory capacity of *P*. *denitrificans* cells

**Fig 1:**
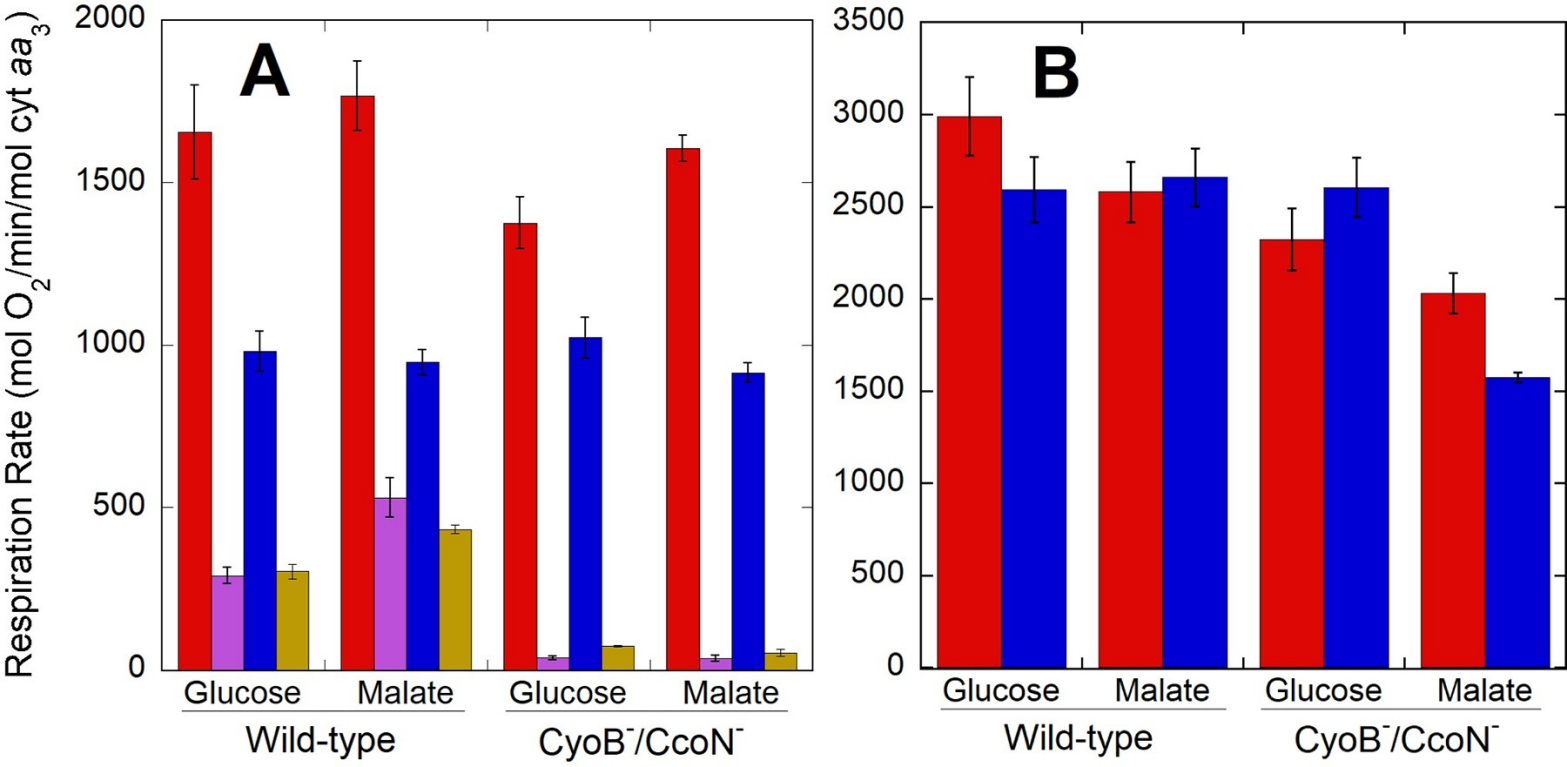
Respiration rates of wild-type and CyoB^-^/CcoN^-^ cells adapted to growth in glucose or malate. Oxygen consumption rates were collected in growth medium in the absence (red) or presence (magenta) of 10 μM antimycin, and in depletion buffer containing no NH_4_^+^ in the absence (blue) or presence (gold) of 10 μM antimycin. Panel A shows normal coupled respiration rates, whereas panel B shows maximal rates obtained after addition of 0.25–0.5 μM of the uncoupler DBNP. Bars correspond to the average ± standard error of 3–8 independent determinations for each strain and condition.

As shown in Fig 1A, *P*. *denitrificans* cells grown on either glucose or malate showed respiratory rates of 1400–1800 mol O_2_/min/mol cyt *aa*_3_. In these experiments, cells were frozen in 15% glycerol upon harvesting and respiratory rates were determined after thawing. Similar rates were measured during culture before harvesting (Table 1). Rates dropped to 900–1000 mol O_2_/min/mol cyt *aa*_3_ when cells respired in medium without NH_4_^+^, the sole nitrogen source in growth medium, reflecting a lower energetic demand when a nitrogen source was lacking. However, since respiration rates in the absence of NH_4_^+^ were 54–74% of those in growth medium, depending on the strain, other energy demanding processes that do not require external nitrogen are predominant in driving oxidative phosphorylation. Maximal respiratory capacity, measured by the addition of the uncoupler 2,6-di-tert-butyl-4-nitrophenol (DBNP), was 2500–3000 mol O_2_/min/mol cyt *aa*_3_ (Fig 1B). An exception was noted for malate-grown cells lacking alternative oxidases, in which inhibition of respiration was observed at saturating uncoupler concentrations. All these experiments were carried out at 30°C, and as expected, respiration increased when determined at 37°C, with observed values in growth medium of ~2800 and ~5000 mol O_2_/min/mol cyt *aa*_3_ for coupled and DBNP uncoupled wild-type cells using either glucose or malate as substrate. This is impressively higher than the maximum respiratory rates of ~700 mol O_2_/min/mol cyt *aa*_3_ estimated for intact mammalian mitochondria [30].

The high respiratory capacity observed in *P*. *denitrificans* was not due to the presence of the additional ubiquinol and cytochrome *c* terminal oxidases in wild-type cells, given that similar respiratory rates were observed when the catalytic subunits of one or both of these alternative oxidases were deleted (Fig 1 and Table 1). Furthermore, in wild-type cells respiring in growth media, the oxygen consumption by the ubiquinol oxidase alternative pathway as determined from the residual respiration in the presence of antimycin, a Complex III inhibitor, corresponded to 17% and 30% of the uninhibited rate in glucose or malate, respectively (Fig 1). Similar absolute rates of antimycin resistant respiration were determined in wild-type cells in the absence of NH_4_^+^. As expected, antimycin inhibited respiration in the CyoB^-^/CcoN^-^ cells by >95% because of the lack of alternative quinol oxidase activity. Growth rate and respiration in growth medium were slightly higher in cells grown in malate compared to glucose (Table 1). However, no difference in respiration as a function of carbon source was observed in medium lacking NH_4_^+^ as a nitrogen source (Fig 1). The higher respiration in the growth medium containing NH_4_^+^ was not due to an increase in cell number, as colony forming units and optical density only increased by ~13% during the first hour of incubation of thawed cells in growth medium (S6 Table). All the subsequent experiments described below were performed in K^+^ depletion buffer lacking NH_4_^+^ to allow more time to record data at higher cell densities before oxygen was depleted from the incubation chamber.

#### Stimulation of respiration by K^+^ replenishment

**Fig 2:**
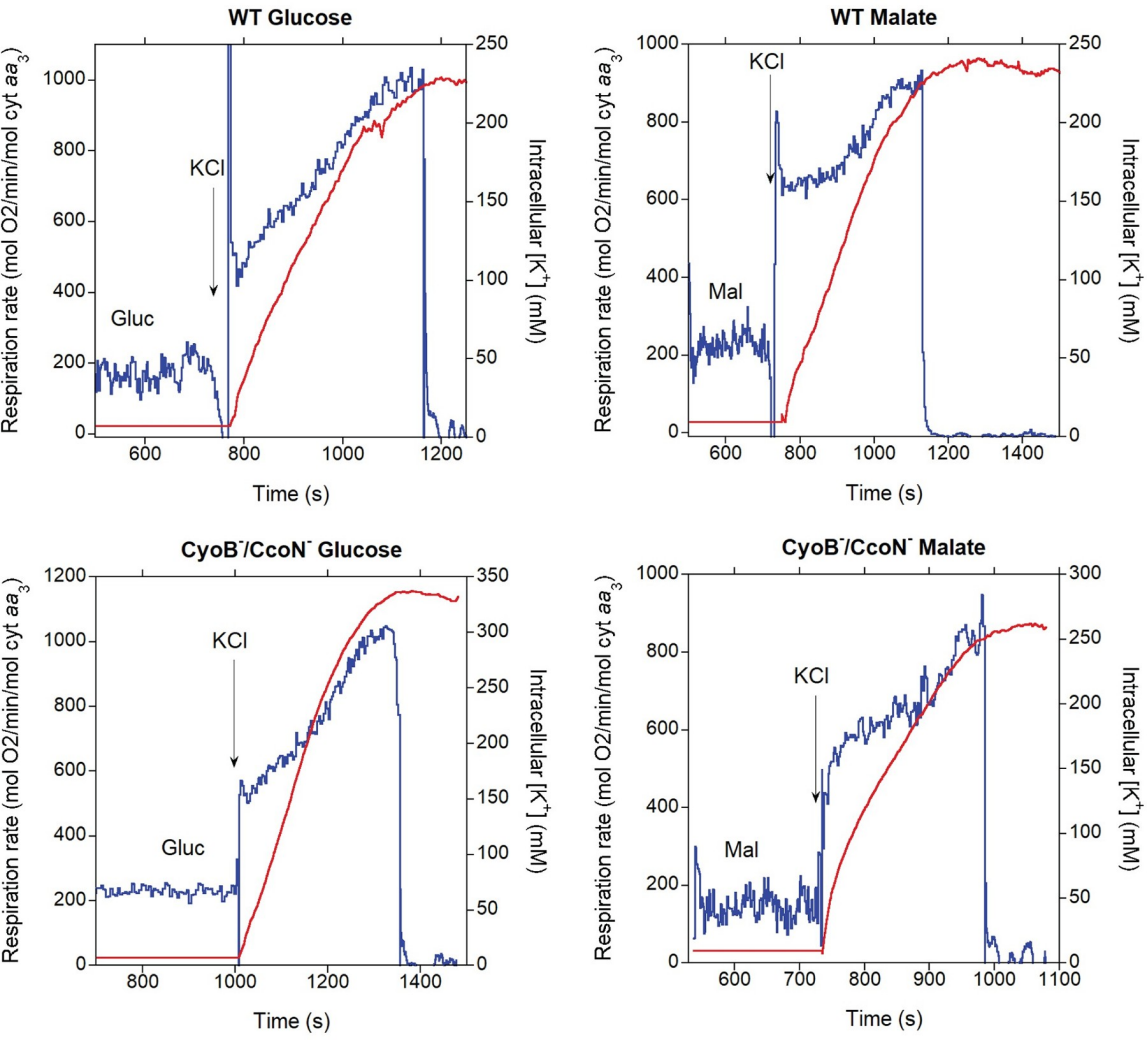
**Respiration rate (blue) and intracellular [K**^**+**^**] (red) before and after addition of 5 mM KCl.** Thawed cells (~2 x 10^9^/ml) of the indicated strains were incubated in depletion NH_4_^+^-free buffer to which 11.1 mM glucose (Gluc; left panel) or 16.5 mM sodium malate (Mal; right panel) was added at ~500 s followed by 5 mM KCl at the time point indicated by the arrow in each panel.

**Fig 3:**
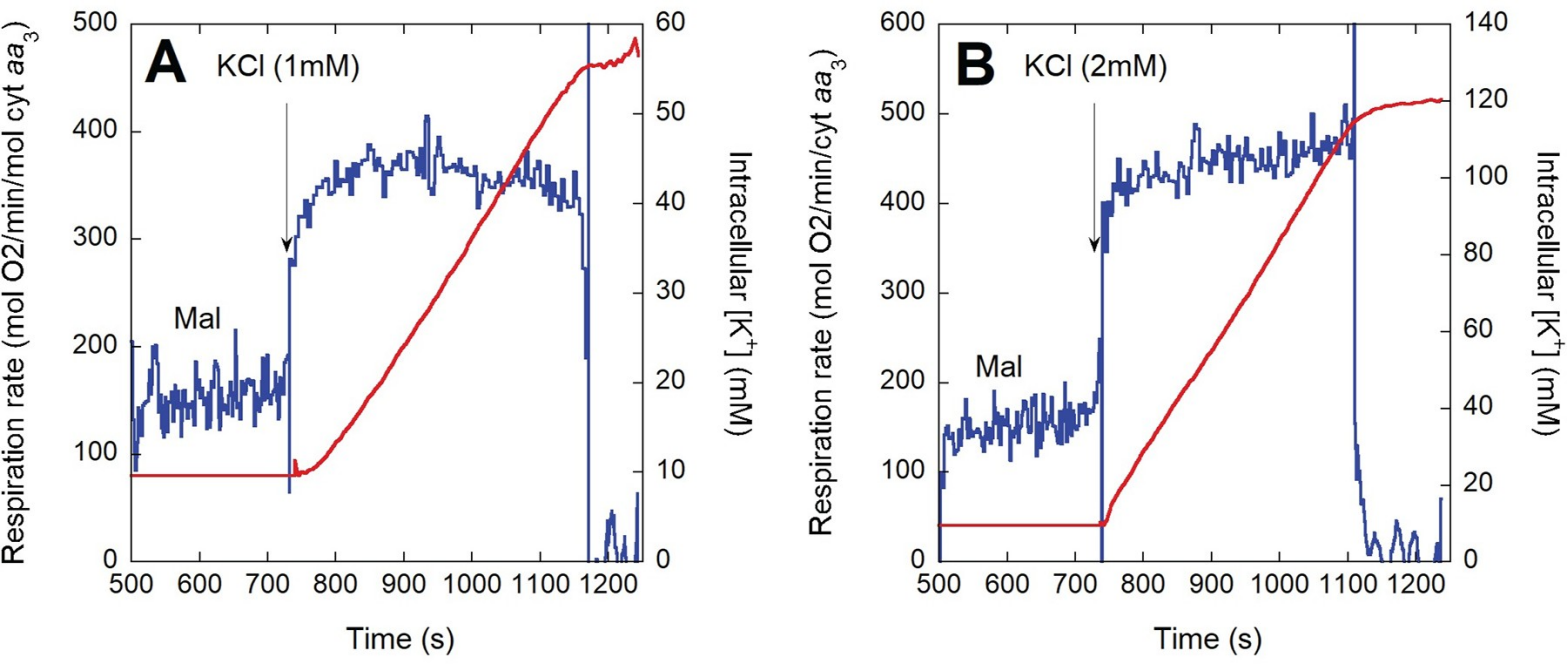
Respiration rate (blue) and intracellular [K^+^] (red) before and after addition of 1 mM (A) or 2 mM (B) KCl to CyoB^-^/CcoN^-^ cells grown in malate. Thawed cells (~2 x 10^9^/ml) were incubated in depletion NH_4_^+^-free buffer to which 16.5 mM sodium malate (Mal) was added at ~500 s followed by KCl at the time point indicated by the arrow in each panel.

In previous work with *P*. *denitrificans*, partial depletion of intracellular K^+^ (by ~40%) was achieved by a growth in low KCl and subjecting cells to a hypotonic shock [21]. This treatment resulted in a stimulation of respiration upon K^+^ replenishment of <4-fold. We found that the hyperosmotic shock resulting from addition of 15% glycerol during harvesting of the cells before freezing resulted in a much more efficient depletion of intracellular K^+^ (>10-fold; see Methods). As shown in the representative oxygen consumption rate and intracellular K^+^ traces of Fig 2, *P*. *denitrificans* cells respired at a slow rate when depleted of K^+^, even after addition of saturating substrate. Upon addition of 5 mM KCl (the same concentration in which the cells were grown), respiration increased within seconds by 2-3-fold. This rapid initial activation occurred before an appreciable increase in intracellular [K^+^] was observed with the K^+^ electrode used, which has a response time of a few seconds. As K^+^ uptake proceeded over the course of 2–3 min, eventually surpassing an intracellular [K^+^] of 200 mM, respiration was stimulated further to a rate ~4-5-fold higher than in the absence of external K^+^. When anoxia was apparently reached, K^+^ uptake stopped but intracellular [K^+^] remained high at least for several minutes, indicating no net leak of [K^+^] in the presence of very low oxygen. We detected a small oxygen leak in our system that sustained a slow residual respiration (see Methods), which could explain the retention of K^+^. As shown in the representative traces of Fig 3, addition of lower KCl concentrations (≤2 mM) evidenced more clearly that the initial rapid activation of respiration occurred before a substantial increase in intracellular [K^+^] was detected. Furthermore, at this lower KCl concentration the slower second phase of respiration activation was not observed in spite of intracellular [K^+^] increasing to almost 120 mM. KCl additions lower than 1 mM could not be accurately evaluated in terms of intracellular [K^+^] due to instability in the K^+^ electrode voltage.

**Fig 4:**
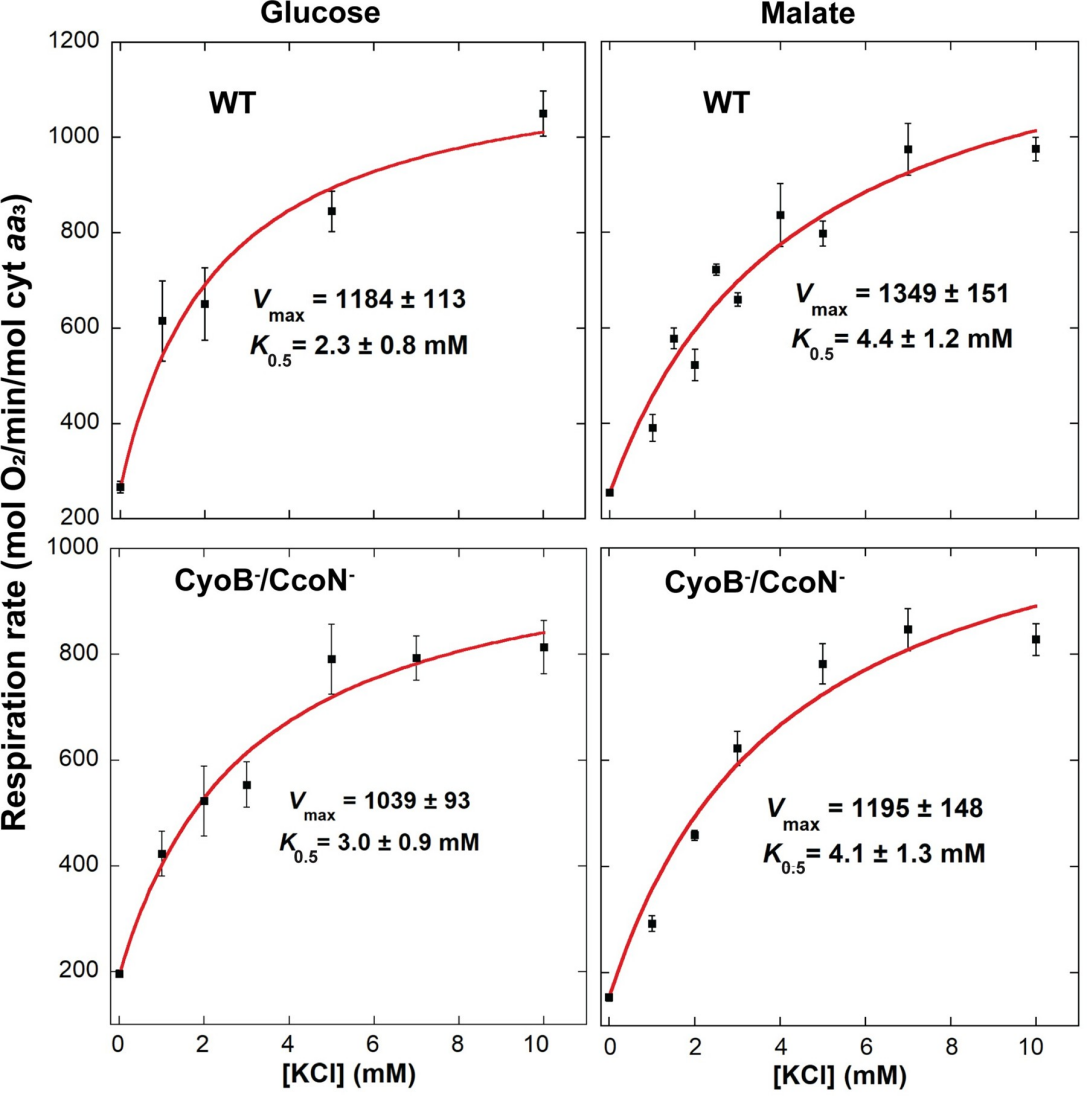
Dependence of respiration rate on the concentration of KCl in wild-type (top) and CyoB^-^/CcoN^-^ (bottom) cells adapted to growth in glucose (left) or malate (right). *V*_max_ and *K*_0.5_ values were calculated by fitting the average respiration at each KCl concentration to a Michaelis-Menten equation with an added offset to account for the respiration observed without KCl present. Error bars correspond to the standard error of 3–8 independent determinations at each KCl concentration, except for 0 mM (n = 27–68) and 10 mM, for which the number of measurements was the same as shown in Table 3.

Table 2 shows the average stimulation of respiration by K^+^ in multiple experiments with each of the different strains tested and cultured either in glucose or malate. Glucose cultured cells exhibited an almost instantaneous stimulation of respiration right after K^+^ addition of almost 3-fold. After several minutes of K^+^ reincorporation, just before oxygen was largely depleted, an almost 4-fold maximal stimulation was attained. Respiration in malate resulted in a 3 to 4-fold immediate increase in respiration rate after KCl addition, which increased to ~4-fold in wild-type cells and almost 6-fold in cells lacking both alternative oxidases (CyoB^-^/CcoN^-^) after several minutes of K^+^ reincorporation. As shown in Fig 4, 10 mM KCl was nearly saturating, generating close to maximal respiratory rates. The concentration necessary for half-maximal activation (*K*_0.5_) of respiration by K^+^ in all strains was in the 2–4 mM range, which is within the range observed in other bacteria that express the low-affinity Trk transport system [31,32]. As shown in Table 3, K^+^ uptake rates after addition of 5 mM KCl were in the range of 2000–3000 mol K^+^/min/mol cyt *aa*_3_ and not significantly different between strains. Uptake rates after addition of >10 mM KCl could not be accurately determined due to the low sensitivity of the K^+^ electrode at higher concentrations.

**Table 2:** Stimulation of respiration by 10 mM KCl in *P*. *denitrificans* strains.

Strain	K^+^ early	K^+^ late	N
WT Glucose	2.68 ± 0.09	3.56 ± 0.16	13
WT Malate	3.18 ± 0.13	3.86 ± 0.18	31
CyoB^-^/CcoN^-^ Glucose	2.91 ± 0.25	4.2 ± 0.22	4
CyoB^-^/CcoN^-^ Malate	4.33 ± 0.32	5.73 ± 0.43	14

Mean ± SE of the fold increase in respiration rate immediately after addition of KCl to a final concentration of 10 mM (K^+^ early) or several minutes later just before oxygen was largely depleted (K^+^ late) with respect to the rate observed with substrate before addition of KCl.

**Table 3:** K^+^ uptake rates upon addition of 5 mM KCl in *P*. *denitrificans* strains.

Strain	K^+^ uptake rate (mol K^+^/min/mol cyt *aa*_3_)	n
WT Glucose	2130 ± 250	7
WT Malate	2980 ± 490	5
CyoB^-^/CcoN^-^ Glucose	2310 ± 250	5
CyoB^-^/CcoN^-^ Malate	2170 ± 140	8

Mean ± SE of the K^+^ uptake rates were calculated from a linear regression of the initial uptake data as shown in Fig 2. A t-test of the data indicated no significant differences (p > 0.17) between strains.

### Membrane potential (ΔΨ) and pH gradient (ΔpH) before and after K^+^ replenishment

**Fig 5:**
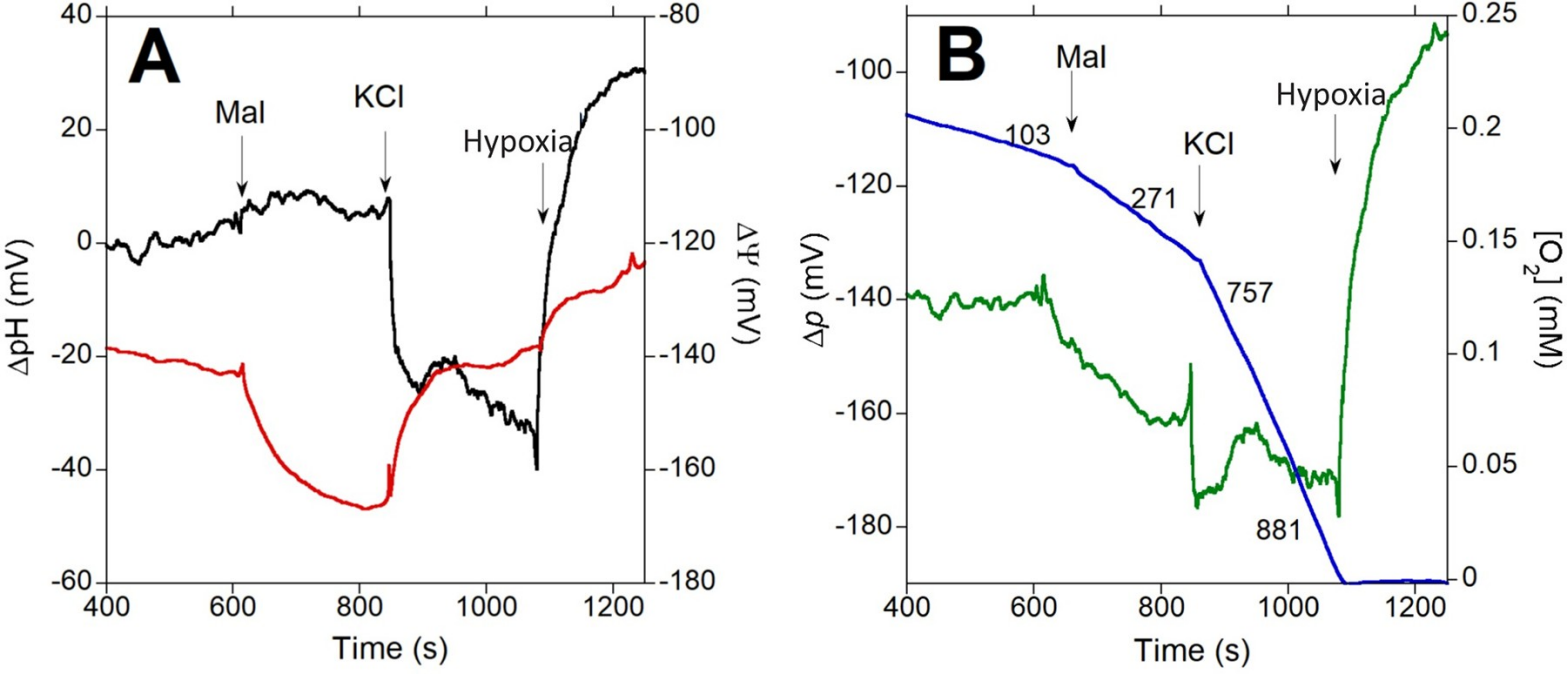
Simultaneous determination of ΔpH and ΔΨ to calculate Δ*p* as a function of respiration rate. 2.5 x 10^9^ K^+^-depleted cells/ml expressing 3 copies of pH-sensitive GFP were incubated in depletion buffer to which 16.5 mM sodium malate (Mal) was added at the indicated time point followed by 10 mM KCl. In panel A, ΔpH (black) and ΔΨ (red) were determined as described in Methods to calculate Δ*p* (green), shown in panel B overlayed with the O_2_ concentration trace (blue) showing respiration rates recorded after the indicated additions.

Positive charges moving into the cytosol of *P*. *denitrificans* cells as K^+^ ions are transported inward could potentially uncouple oxidative phosphorylation and result in a higher respiratory rate. Indeed, as shown in the representative trace of Fig 5A, K^+^ addition resulted in a fast >20 mV depolarization of ΔΨ upon addition of 10 mM K^+^. However, an increase in the magnitude of ΔpH was also observed at the same time, which was likely catalyzed by the K^+^/H^+^ antiporter system. Care must be taken in interpreting the time courses of ΔΨ and ΔpH. The optical pH measurement responds rapidly to changes intracellular pH, while the tetraphenylphosphonium (TPP^+^) electrode needs the concentration of the reporting ion in the large extracellular volume to be modified in response to its redistribution across the bacterial membrane. This difference in response time between the ΔΨ and ΔpH measurements could explain the slight and transient net increase in Δ*p* immediately after KCl addition (Fig 5B). While respiration increased ~3-fold, relatively small (<10 mV) opposing changes in ΔΨ and ΔpH were observed in the next few minutes before oxygen became largely depleted (Fig 5A and 5B). At hypoxia, ΔpH completely collapsed and became slightly inverted (more acidic inside the cell) while ΔΨ was depolarized by ~20 mV. As shown in Table 4, averaging the ΔΨ and ΔpH values observed across numerous experiments between 2 minutes after KCl addition and right before oxygen was largely depleted allowed to calculate that Δ*p* increased by 10 mV after K^+^ replenishment. Since Δ*p* (and not only ΔΨ) is the actual product of the proton pumping activity of respiratory complexes, K^+^ activation of oxidative phosphorylation cannot be explained by uncoupling due to K^+^ transport into the cell. It is also noteworthy that ΔΨ was still -115 mV on average when oxygen was almost zero (Table 4). This explains why K^+^ was retained because the ΔΨ at this very low oxygen concentration was sufficient to maintain a gradient nearly two orders of magnitude higher inside the cells.

**Table 4:** Membrane potential (ΔΨ), pH gradient (ΔpH) and proton motive force (Δ*p*) quantitation in malate-grown *P*. *denitrificans* cells.

Experimental condition	ΔΨ (mV)	ΔpH (mV)	Δ*p* (mV)	Respiration rate (mol O_2_/min/mol cyt *aa*_3_)
No addition	-125.7 ± 3.8	-10.5 ± 2.5	-136.2 ± 3.1	83.3 ± 12.4
+ Malate	-154 ± 2.9	-4.9 ± 2.3	-158.9 ± 2.4 ^a^	222.8 ±12.3
+ KCl	-129.7 ± 3.1	-39.3 ± 3.5	-169.0 ± 3.0 ^a^	847.1 ± 22.1^b^
Hypoxia	-115.0 ± 3.4	9.9 ± 2.7	-105.1 ± 3.1	0 ± 0
Nigericin	-100.6 ± 3.4	1.0 ± 0.7	-99.6 ± 3.1	0 ± 0

Mean ± SE of 10 independent determinations. Values were determined between 2 min after addition of 10 mM KCl and before oxygen was largely depleted.

^a^Δ*p* was statistically different between the + Malate and the + KCl conditions (p = 0.0003) according to a paired t-test.

^b^Maximal rate determined >2 min after addition of 10 mM KCl. Respiration rate within 1 min after KCl addition was 676.8 ± 23.2 mol O_2_/min/mol cyt *aa*_3_.

### Effect of the inhibition of ATP synthesis on respiration, ΔΨ, ΔpH, and Δ*p* upon K^+^ addition

**Fig 6:**
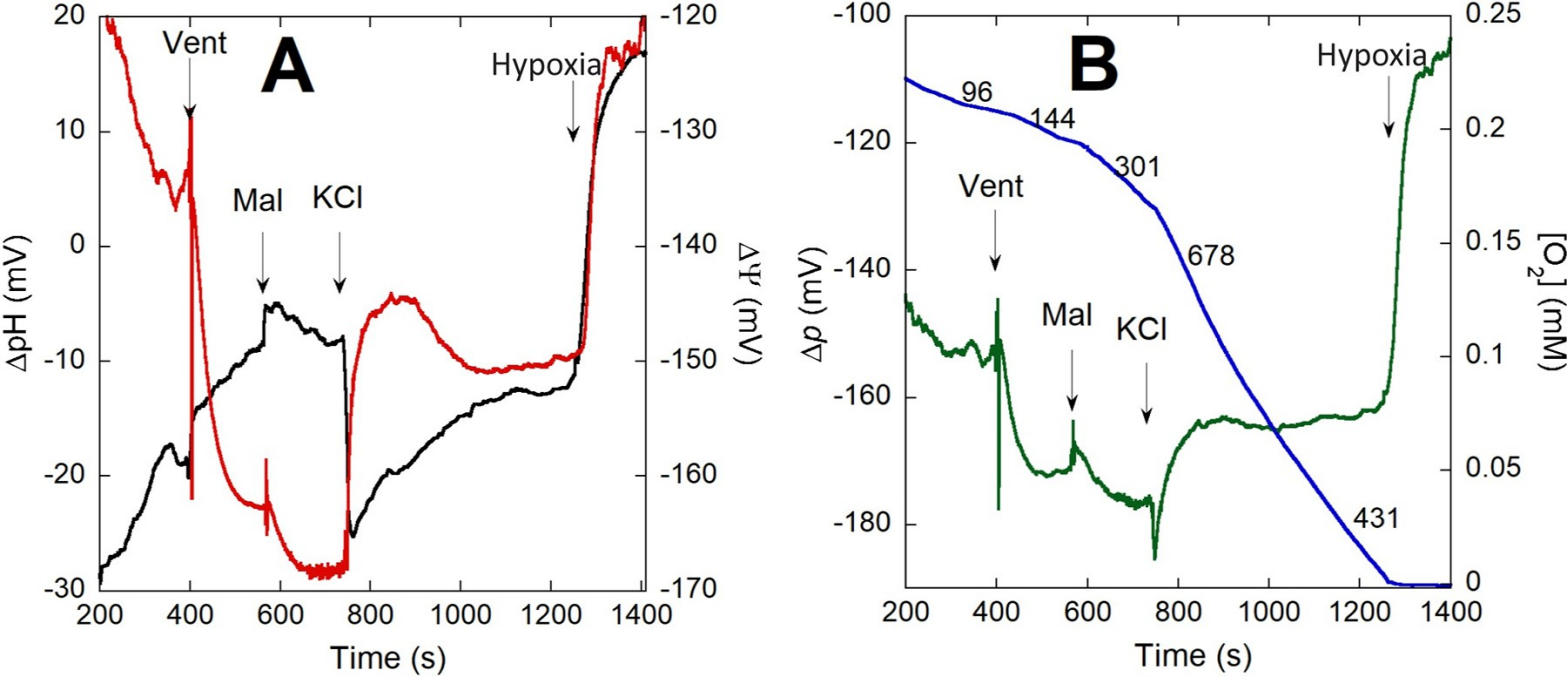
Simultaneous determination of ΔpH and ΔΨ to calculate Δ*p* as a function of respiration rate in the presence of venturicidin. 2 x 10^9^ K^+^-depleted cells/ml expressing 3 copies of pH-sensitive GFP were incubated in depletion buffer in the presence of 10 μM venturicidin (Vent) for 3 min before 16.5 mM sodium malate (Mal) was added at the indicated time point followed by 10 mM KCl. In panel A, ΔpH (black) and ΔΨ (red) were determined as described in Methods to calculate Δ*p* (green), shown in panel B overlayed with the O_2_ concentration trace (blue) showing respiration rates recorded after the indicated additions.

In order to determine the consequences of inhibiting ATP synthesis by oxidative phosphorylation on the effects of re-exposure to K^+^, we incubated *P*. *denitrificans* cells with venturicidin, which blocks the movement of H^+^ through Complex V [33,34]. As shown for a representative experiment in Fig 6, venturicidin did not prevent the >20 mV depolarization of ΔΨ observed immediately after KCl addition, consistent with an initial electrogenic K^+^ uptake. ΔpH also increased very rapidly but did not compensate completely for the depolarization of ΔΨ, resulting in a net loss of ~10 mV in Δ*p*. Respiration rate increased >2-fold for the first 2–3 min after KCl addition, but decreased by ~40% as ΔΨ partially repolarized. ΔpH slowly decreased until becoming stable before hypoxia, resulting in a nearly constant Δ*p* throughout the period following KCl addition. Addition of venturicidin after KCl resulted in a complete repolarization of ΔΨ, but still inhibited respiration only partially (see representative trace of S1 Fig). The summarized data in Table 5 for the average of 3–6 experiments shows that venturicidin acted rapidly after addition to the cells as evidenced by the hyperpolarization of ΔΨ by ~27 mV, consistent with a blockage of H^+^ back flux through Complex V. Although ATP synthesis was already blocked, respiration still increased 2.3-fold during the first 2 min after KCl addition, primarily driven by electrogenic K^+^ influx. The fast 21 mV depolarization of ΔΨ driven by the initial K^+^ influx was compensated immediately by an almost equivalent increase in ΔpH, with no statistically significant decrease in Δ*p*. A slight repolarization of ΔΨ by ~9 mV and a decrease in respiration rate occurred with no change in Δ*p*. Comparing the steady state respiration rate just before hypoxia in the absence of venturicidin (850 mol O_2_/min/mol cyt *aa*_3_, see Table 4) with that in the presence of the inhibitor (450 mol O_2_/min/mol cyt *aa*_3_, Table 5), an almost 2-fold difference in rate was observed. This occurred in spite of Δ*p* being only ~6 mV less favorable for H^+^ pumping by the respiratory chain when ATP synthesis was allowed to proceed.

**Table 5:** Membrane potential (ΔΨ), pH gradient (ΔpH) and proton motive force (Δ*p*) quantitation in malate-grown *P*. *denitrificans* cells in the presence of 10 μM venturicidin.

Experimental condition	ΔΨ (mV)	ΔpH (mV)	Δ*p* (mV)	Respiration rate (mol O_2_/min/mol cyt *aa*_3_)
No addition	-130 ± 3.4	-15.4 ± 2.6	-145.3 ± 7.6	167.5 ± 17
+ Venturicidin	-156.9 ± 2.4	-7.9 ± 3.4	-164.8 ± 5.1	152 ± 4.6
+ Malate	-163.1 ± 2.3	-7.4 ± 3	-170.5 ± 3.6	311.9 ± 12.6
+ KCl (first phase)^a^ (second phase)^b^	-142 ± 1.6	-21.4 ± 3	-163.5 ± 1.8	721.2 ± 36.4
	-151.4 ± 1.9	-11.3 ± 4	-162.6 ± 1.5	453.1 ± 24.3
Hypoxia	-131.6 ± 2.9	17.9 ± 3.9	-113.7 ± 5.6	0 ± 0
+ Nigericin	-99.7 ± 6.5	3.3 ± 1.4	-96.4 ± 7.7	0 ± 0

Mean ± SE of 6 (ΔΨ and respiration rate) or 3 (ΔpH and Δ*p*) independent determinations. Reagents were sequentially added as shown in Fig 6. Δ*p* was not statistically different between the + Malate and the + KCl conditions (p = 0.13) according to a paired t-test.

^a^The first phase corresponds to the average of the first 2 min after KCl addition.

^b^Values for the second phase correspond to the average between 4 min after KCl addition and just before hypoxia.

### Effect of K^+^ replenishment on the redox state of oxidative phosphorylation complexes

**Fig 7:**
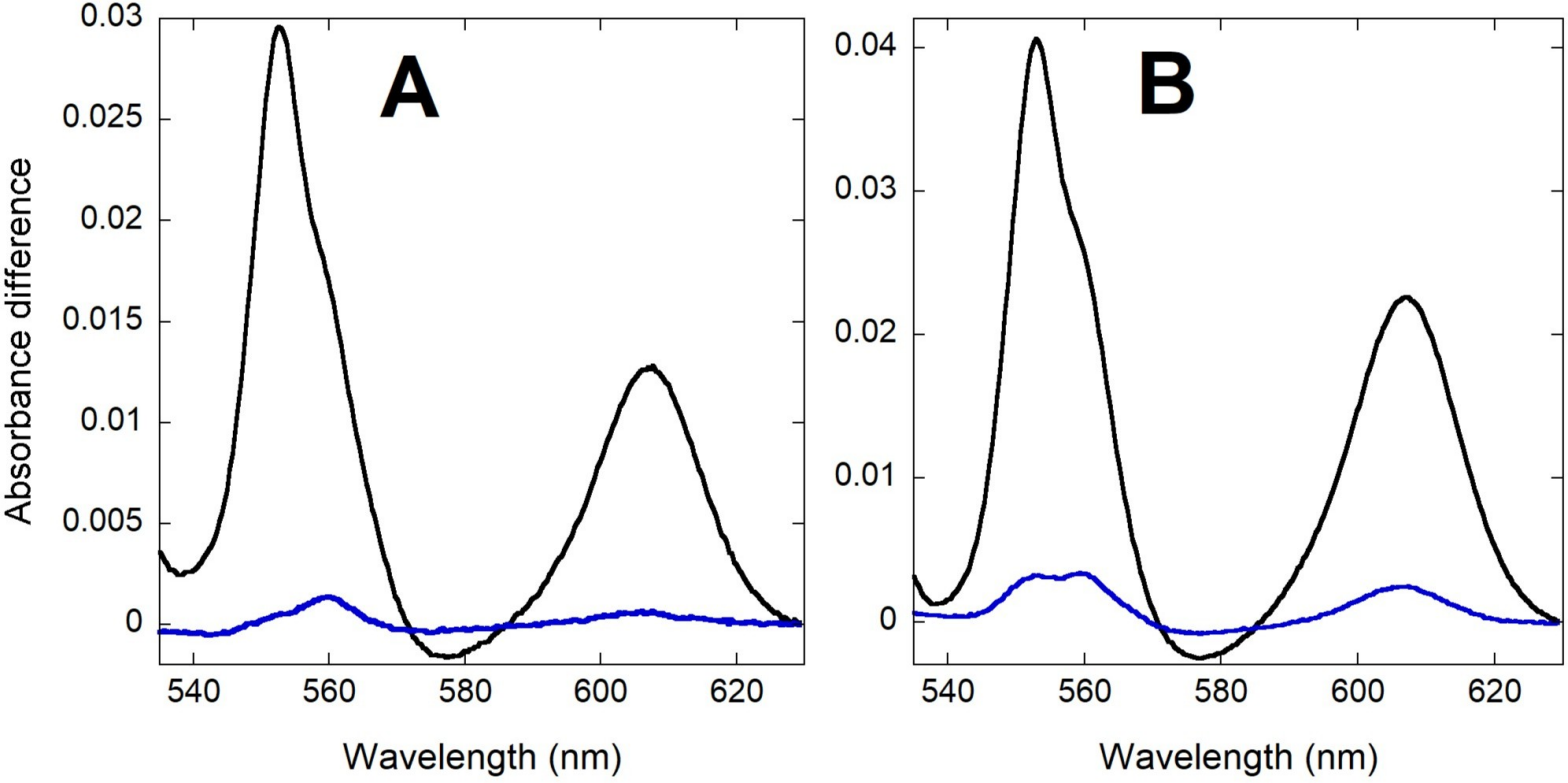
**Comparison of the magnitude of cytochrome absorbance changes induced by the addition of KCl (blue) relative to the full absorbance scale observed at anoxia (black).** CyoB^-^/CcoN^-^ K^+^-depleted cells respiring on glucose (A; 3.25 x 10^10^ cells/ml) or sodium malate (B; 5.41 x 10^10^ cells/ml) were used to collect difference spectra calculated relative to incubation before substrate. 400 spectra collected every 0.5 s were averaged for each condition and the resulting averaged spectra were subtracted as indicated (KCl-substrate or anoxia-substrate).

**Fig 8:**
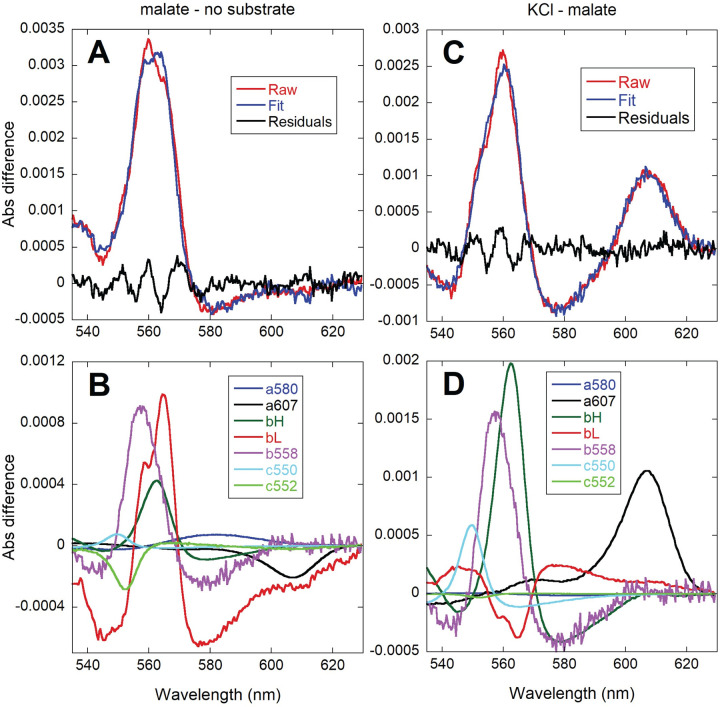
Comparison of the absorbance changes induced by the addition of 16.5 mM sodium malate (left, A and B) and 20 mM KCl after malate (right, C and D). Difference spectra of 8.4 x 10^9^/ml CyoB^-^/CcoN^-^ K^+^ depleted cells were calculated relative to incubation without substrate (left) or relative to the steady state after malate addition (right). 200 spectra collected every 0.5 s were averaged for each condition and the resulting averaged spectra were subtracted as indicated (malate-substrate or KCl-malate). The top panels (A and C) show the fit (blue) of the raw spectral data (red) and the residuals of the fit (green). The bottom (B and D) panels show the contribution of each cytochrome reference to the fitted spectra.

In order to characterize the potential target sites for the activation of respiration by K^+^ addition, we analyzed the redox levels of chromophores in *P*. *denitrificans* by integrating sphere absorbance spectroscopy, as we have previously reported for isolated mitochondria [35,36]. These experiments were performed in the strain lacking both alternative terminal oxidases. As shown in Fig 7, the observed increase in absorbance of the *b*, *c* and *a*-type cytochromes upon K^+^ addition was <10% of the total dynamic range determined at hypoxia. Given that respiration increased by 4-6-fold (see Table 2), the relatively small changes in the steady-state electron residence within the respiratory chain chromophores were consistent with a parallel activation of metabolic reactions that feed electrons into the respiratory chain as well as energy consuming reactions, which agrees with the conservation of Δ*p*, as described above. Furthermore, a differential effect of K^+^ on respiratory complexes was evidenced when accelerating respiration by either substrate or K^+^ addition, as shown in the representative spectra of Fig 8. The respiratory rate upon malate addition increased ~3-fold (Table 4) relative to the no substrate incubation condition, and as shown in Fig 8A and 8B, was associated with an increase in the reduction of all three cytochrome *b* species (*b*558, tentatively assigned to succinate dehydrogenase, as well as the *b*_H_ and *b*_L_ hemes of Complex III). The larger increase in *b*_L_ reduction relative to *b*_H_, agrees well with the observed hyperpolarization of ΔΨ observed upon malate addition (see Fig 5 and Table 4). These two hemes are located close to opposite sides of the membrane, and the electron distribution between them is known to be sensitive to changes in ΔΨ [37]. In contrast, *c*-type and *aa*_3_ cytochromes showed little redox change, with some of them (the membrane-bound *c*552 and the Complex IV-related *a*607 component) decreasing slightly in their absorbance in spite of a higher respiratory rate. When KCl was added to the malate respiring cells (Fig 8C and 8D), an extra reduction of *b*558 and *b*_H_ was observed while *b*_L_ became partly oxidized, consistent with the slight depolarization of ΔΨ observed upon K^+^ replenishment (see Fig 5 and Table 4). In this case, cytochrome *c*550 and *a*607 absorbance increased, which is the pattern expected from the >4-fold stimulation of respiration according to previous studies in *P*. *denitrificans* membranes [38] and isolated mitochondria [35,36]. The difference in the pattern of cytochrome *a*607 absorbance observed between substrate only and KCl addition (in spite of a similar relative increase in respiration) suggested a very different electron distribution within Complex IV induced by the presence of K^+^.

**Fig 9:**
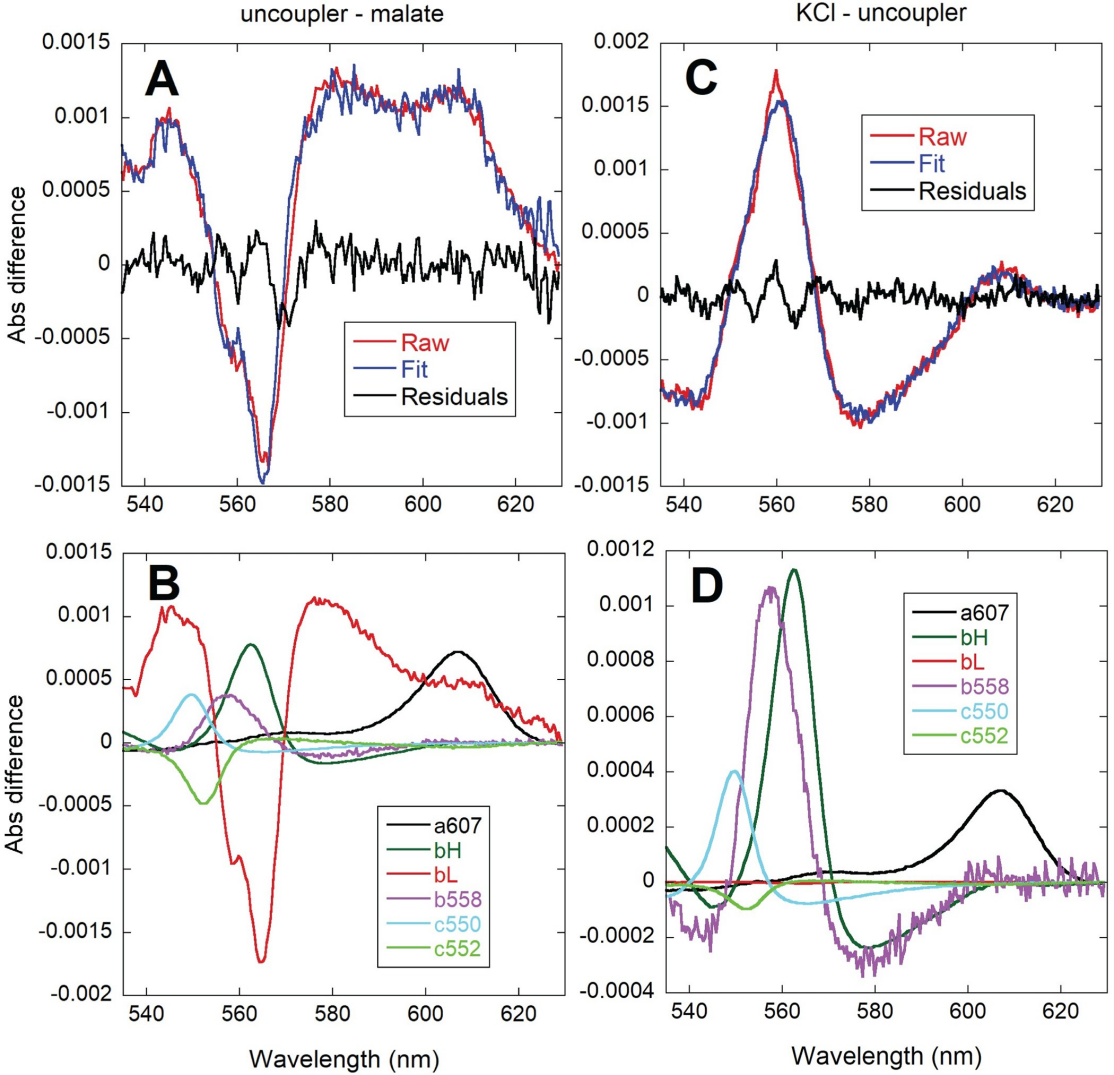
Comparison of the absorbance changes induced by the addition of 3 μM DBNP after 16.5 mM sodium malate (left, A and B) and 20 mM KCl after DBNP (right, C and D). Difference spectra of 8.4 x 10^9^/ml CyoB^-^/CcoN^-^ K^+^ depleted cells were calculated relative to the steady state after sodium malate addition (left) or relative to the steady state after uncoupler addition (right). 200 spectra collected every 0.5 s were averaged for each condition and the resulting averaged spectra were subtracted as indicated (uncoupler-malate or KCl-uncoupler). The top panels (A and C) show the fit (blue) of the raw spectral data (red) and the residuals of the fit (green). The bottom panels (B and D) show the contribution of each cytochrome reference to the fitted spectra.

As shown in Fig 9A and 9B, the spectral change induced by a subsaturating concentration of the uncoupler DBNP was dominated by a strong oxidation of the *b*_L_ heme and a slight increase in *a*607. This pattern was very different from those observed by either the addition of malate or KCl (compare to the spectra in Fig 8). This implied that the increase in respiration induced acutely by K^+^ was not due only to the partial dissipation of ΔΨ because of the transport of positive charges into the cell. Moreover, addition of K^+^ after uncoupler (Fig 9C and 9D) resulted in increases in the absorbance of *b*558, *b*_H_, *c*550 and *a*607, albeit to a lower extent than in the absence of uncoupler. This indicated that K^+^ replenishment was still able to activate electron input into the respiratory chain as well as redistribute electrons within Complex IV, even when the presence of uncoupler had already partially dissipated ΔΨ.

### Changes in concentrations of metabolites in the presence of K^+^

As shown in Table 6, the concentration of several glycolytic intermediates increased after a 20 min incubation of wild-type cells in the presence of either glucose or malate as carbon source together with 10 mM KCl compared to incubation with substrate only. For example, glucose 6-phosphate increased ~4 and 6-fold in the presence of K^+^ with glucose or malate, respectively, whereas fructose 6-phosphate was only detected when K^+^ was present in the incubation mixture (except for one of the five malate samples). This was also observed for 3-phosphoglycerate, dihydroxy acetone phosphate and glycerol-3-phosphate in glucose respiring cells. 6-phosphogluconate, which can be generated by glycolysis through the Entner-Doudoroff pathway, was also only detectable in the presence of K^+^ when using malate as substrate. In contrast, glucose 1-phosphate, which is an intermediate between glycolysis and both glycogenesis and glycogenolysis, was only detectable in the absence of K^+^. In malate incubated cells, glycogen decreased by ~40% in the presence of K^+^ and glucose increased by ~1 mM, an amount equivalent to ~80% of the hydrolyzed glycogen (S2 Fig). Thus, even in the absence of external glucose, a general activation of glucose utilization by K^+^ was evident by an increased glucose availability and an activation of glycolytic enzymes. Surprisingly, lactate concentrations under all conditions was very high (6–7 mM) even though *P*. *denitrificans* cells lack the NAD-dependent lactate dehydrogenase linked to glycolysis [39]. The lactate concentrations reported from the metabolomic analysis were confirmed by two other independent methods: short-chain fatty acid reverse chromatography [40] and by HPLC-mass spectrometry [41]. In the case of TCA cycle intermediates, with both glucose and malate, the concentration of citrate was 6 to 7-fold higher when K^+^ was present while 2-oxoglutarate increased from almost undetectable levels in the absence of K^+^ (found at <1 mM in only one of the malate respiring samples) to almost 5 mM with K^+^. A similar observation was made for succinate in cells using glucose, increasing from <0.6 mM without K^+^ (detected in only one sample) to ~2 mM.

**Table 6:** Concentration of metabolites of glycolysis and the TCA cycle in wild-type cells in the absence or presence of 10 mM KCl^a^.

Metabolite	Concentration (pmol/10^9^ cells)^b^				Ratio^c^	
	Glucose	Glucose + K^+^	Malate	Malate + K^+^	Glucose K^+^/no K^+^	Malate K^+^/no K^+^
**Glycolysis:**						
Glucose 1-phosphate	113 ± 13	N.D.	75 ± 6	N.D.	low	low
Glucose 6-phosphate	198 ± 47	759 ± 93	93 ± 8	558 ± 20	3.83	6.02
Fructose 6-phosphate	N.D.	141 ± 5	77	132 ± 6	high	1.71
Fructose 1,6-diphosphate	514 ± 134	468 ± 60	528 ± 103	113 ± 11	0.91	0.21
3-Phosphoglyceric acid	N.D.	781 ± 56	N.D.	N.D.	high	N.D.
Dihydroxyacetone phosphate	N.D.	414 ± 73	107	N.D.	high	Low
Glycerol 3-phosphate	N.D.	201 ± 30	111 ± 12	78 ± 8	high	0.71
6-Phosphogluconic acid	N.D.	N.D.	N.D.	213 ± 28	N.D.	High
Lactic acid	6500 ± 650	5850 ± 1310	5400 ± 1,040	7,020 ± 380	0.90	1.3
**TCA Cycle:**						
Acetyl CoA	113 ± 22	61 ± 8	22	N.D.	0.54	low
Citric acid	338 ± 59	2170 ± 180	383 ± 66	2900 ± 270	6.41	7.58
2-Oxoglutaric acid	N.D.	4830 ± 970	714	4420 ± 830	high	6.17
Succinic acid	513	1750 ± 640	2250 ± 250	4010 ± 750	3.40	1.79
Fumaric acid	N.D.	N.D.	3050 ± 840	3770 ± 1100	N.D.	1.23
Malic acid	691 ± 102	755 ± 156	51200 ± 5200	53300 ± 4100	1.09	1.04

^a^Metabolites with statistically significant differences (p < 0.05; Welch’s t-test) between the presence and absence of K^+^ in at least one substrate (glucose or malate) are shaded orange, as are the respective concentration values and ratios.

^b^Mean ± SE of 3–5 experiments. N.D. = below detection limit. Metabolite concentration can also be expressed in μM assuming an intracellular volume of 1 μl/10^9^ cells.

^c^ A “high” or “low” ratio indicates that the metabolite was not detected in the substrate only or in the substrate + K^+^ condition, respectively, but was detected in the other condition.

Table 7 shows that the concentration of several amino acids and related compounds changed when comparing cells incubated in substrate with and without K^+^. Although the medium did not contain a nitrogen source, substantial changes occurred in amino acid levels. The cells may have obtained the required nitrogen by transamination, by hydrolysis of other amino acids, or by catabolism of nucleic acids, especially ribosomal RNA [42]. Glutamate concentration was the highest of all amino acids (>2 mM) and it doubled in glucose respiring cells in the presence of K^+^. In contrast, glutamine, present at 0.4–0.5 mM in the substrate only condition, decreased by 88% (in glucose cells) or 96% (in malate cells) when K^+^ was included, suggesting a direct or indirect K^+^-dependent activation of the transfer of its amide group. Choline, another potential source of nitrogen, decreased by ~80–90% in the presence of K^+^ in either substrate. The concentration of 2-oxoisovalerate, which is linked to valine synthesis or degradation by transamination, was as high as valine in the presence of K^+^ but below detection limits in its absence regardless of substrate. Lysine concentration also decreased by 65–80% with K^+^ present depending on the substrate, whereas a more modest decrease of ~30% was observed for arginine. The polyamine spermidine increased ~2-fold in the presence of K^+^ in glucose respiring cells.

**Table 7:** Concentration of metabolites involved in amino acid metabolism in wild-type cells in the absence or presence of 10 mM KCl^a^.

Metabolite	Concentration (pmol/10^9^ cells)^b^				Ratio^c^	
	Glucose	Glucose + K^+^	Malate	Malate + K^+^	Glucose K^+^/no K^+^	Malate K^+^/no K^+^
Alanine	325 ± 17	273 ± 40	269 ± 32	260 ± 28	0.84	0.96
Arginine	298 ± 7	204 ± 19	259 ± 10	183 ± 15	0.68	0.71
Asparagine	16 ± 1	14 ± 3.3	16 ± 2.5	15 ± 2	0.86	0.98
Aspartic acid	86 ± 6	68 ± 14	110 ± 19	98 ± 11	0.79	0.89
Glutamine	456 ± 21	57 ± 15	381 ± 25	16 ± 2.8	0.12	0.04
Glutamic acid	2200 ± 100	4470 ± 390	2880 ± 60	3320 ± 280	2.03	1.15
Glycine	664 ± 242	468 ± 64	392 ± 64	460 ± 49	0.70	1.17
Histidine	36 ± 4.2	27 ± 4	29 ± 8	30 ± 6	0.76	1.06
Isoleucine	37 ± 2.5	33 ± 7	32 ± 4.3	34 ± 4.1	0.91	1.05
Leucine	57 ± 2.8	61 ± 12	49 ± 5	55 ± 6	1.07	1.12
Lysine	304 ± 30	62 ± 9	165 ± 20	56 ± 5	0.20	0.34
Methionine	16 ± 1.8	8.6 ± 1.8	13 ± 3.6	9.3 ± 1.3	0.52	0.72
Phenylalanine	36 ± 1.6	82 ± 7	37 ± 2.4	37 ± 3.5	2.28	1.00
Proline	66 ± 4	195 ± 20	84 ± 7	186 ± 13	2.96	2.21
Serine	408 ± 29	366 ± 85	440 ± 84	449 ± 59	0.9	1.02
Threonine	120 ± 9	99 ± 16	108 ± 12	105 ± 13	0.82	0.97
Tryptophan	7.7 ± 1.1	5.8 ± 1.3	4.8 ± 0.7	4.2 ± 0.6	0.75	0.89
Tyrosine	30 ± 2.8	41 ± 3.2	24 ± 3	31 ± 2.2	1.36	1.28
Valine	157 ± 5	232 ± 25	102 ± 10	156 ± 9	1.48	1.53
2-Oxoisovaleric acid	N.D.	250 ± 22	N.D.	177 ± 15	high	high
Citrulline	13 ± 5.8	32 ± 3.6	9.3 ± 1.5	16 ± 4	2.41	1.69
Ornithine	140 ± 63	136 ± 25	116 ± 21	144 ± 15	0.97	1.24
Putrescine	104 ± 47	112 ± 13	92 ± 19	105 ± 20	1.08	1.13
Spermidine	314 ± 140	585 ± 78	267 ± 40	286 ± 30	1.87	1.07
Creatine	48 ± 21	21 ± 10	17 ± 14	21 ± 4	0.43	1.22
Creatinine	3.3 ± 0.3	3.8 ± 0.5	3.3 ± 0.4	4.4 ± 0.9	1.16	1.30
GABA	2.6 ± 0.08	5.0 ± 0.4	3.3 ± 0.3	4.7 ± 0.7	1.94	1.44
Anthranilic acid	1.4	2.3 ± 0.3	14 ± 0.8	5.2 ± 0.7	1.64	0.38
β-Ala	5.7 ± 0.2	11 ± 1.4	4.9 ± 0.4	13 ± 2.1	1.93	2.65
Betaine	110 ± 20	150 ± 32	36 ± 0.004	73 ± 18	1.36	2.00
Choline	162 ± 11	20 ± 4.1	122 ± 10	27 ± 3.7	0.12	0.22
*S*-Adenosylmethionine	16 ± 1.7	33 ± 2.1	8.3 ± 0.9	25 ± 2.3	2.07	2.98
Glutathione (GSSG)	136 ± 4	178 ± 7	65 ± 5	57 ± 5	1.31	0.88
3-Hydroxybutyric acid	N.D.	110 ± 4.5	231 ± 27	161 ± 28	high	0.70

^a^Metabolites with statistically significant differences (p < 0.05; Welch’s t-test) between the presence and absence of K^+^ in at least one substrate (glucose or malate) are shaded orange, as are the respective concentration values and ratios.

^b^Mean ± SE of 3–5 experiments. N.D. = below detection limit. Metabolite concentration can also be expressed in μM assuming an intracellular volume of 1 μl/10^9^ cells.

^c^ A “high” ratio indicates that the metabolite was not detected in the substrate only condition.

The concentration of ATP, GTP, UTP, and to a lesser degree that of their corresponding diphosphates, was significantly higher in the presence of K^+^ than in its absence (Table 8). ATP increased 1.8 (in malate) or 2.7-fold (in glucose), whereas GTP increased ~3-fold with either substrate, and UTP was 3 to 4.7-fold higher depending on the substrate. The increase of ADP in the presence of K^+^ was more modest (~40%) than that of GDP (~2-fold), while UDP increased by ~60% in glucose respiring cells and was only detected when K^+^ was present with malate as substrate. However, the ADP and UDP concentrations must be interpreted cautiously, since their tight binding or even low levels of ATP and GTP hydrolysis could introduce errors in their measured levels. For this study we decided to also determine the critical adenine nucleotides and GTP concentrations by HPLC/UV quantitation (values in parentheses in Table 8). Concentrations of AMP, ADP, and GTP were very similar with both methodologies under most conditions. Although ATP concentrations were 55–85% higher compared to capillary electrophoresis/mass spectrometry values, a similar relative increase was found upon K^+^ replenishment. This result indicates that the increase in respiratory rate observed upon K^+^ replenishment was not due to depletion of high energy phosphate metabolites associated with higher ATP hydrolysis rates, as is the classical model for respiratory control in isolated mitochondria preparations. The total concentration of adenine nucleotides increased between 1.7 and 2.3-fold, while that of guanidine and uridine nucleotides nearly tripled, likely due to increased RNA breakdown [42] or *de novo* synthesis in the presence of K^+^.

**Table 8:** Concentration of nitrogenated bases, nucleosides and nucleotides in wild-type cells in the absence or presence of 10 mM KCl^a^.

Metabolite	Concentration (pmol/10^9^ cells)^b^				Ratio^c^	
	Glucose	Glucose + K^+^	Malate	Malate + K^+^	Glucose K^+^/no K^+^	Malate K^+^/no K^+^
Adenine	21 ± 1.4	21 ± 1.1	17 ± 1.9	17 ± 1	1.01	0.98
Adenosine	12 ± 2.9	9.1 ± 0.6	6.7 ± 0.2	5.8 ± 0.5	0.76	0.87
AMP^d^	76 ±25 (109 ± 10)	106 ± 83 (103 ± 36)	57 ± 7 (81 ± 8)	67 ± 5.2 (85 ± 3)	1.39 (0.96)	1.16 (1.06)
ADP^d^	186 ± 15 (227 ± 18)	264 ± 52 (241 ± 18)	140 ± 4.4 (178 ± 4)	200 ± 6 (105 ± 7)	1.42 (1.06)	1.43 (0.59)
ATP^d^	623 ± 78 (1079 ± 116)	1690 ± 90 (2620 ± 118)	451 ± 21 (834 ± 39)	808 ± 75 (1346 ± 133)	2.72 (2.43)	1.79 (1.61)
Guanine	25 ± 2.5	27 ± 1.7	21 ± 2.4	22 ± 1.7	1.10	1.05
Guanosine	4.9 ± 0.6	6.1 ± 0.5	5.4 ± 0.4	4.4 ± 0.4	1.25	0.82
GDP	93 ± 7	165 ± 38	64 ± 5.2	144 ± 9	1.78	2.24
GTP	429 ± 45 (404± 48)	1,427 ± 112 (1401 ± 65)	288 ± 13 (303± 14)	815 ± 84 (819± 80)	3.33 (3.47)	2.83 (2.7)
Cytosine	27 ± 2.5	28 ± 0.9	28 ± 1.1	26 ± 1.7	1.05	0.93
Cytidine	3 ± 0.3	6.4 ± 0.6	2.7 ± 0.2	4.2 ± 0.3	2.16	1.52
CTP	307 ± 52	397 ± 23	235 ± 28	162 ± 12	1.29	0.69
Thymine	587 ± 52	572 ± 44	82 ± 29	45 ± 7	0.97	0.55
Thymidine	26 ± 3.4	26 ± 2.4	26 ± 2.6	22 ± 1.8	1.00	0.83
Uracil	45 ± 11	49 ± 2.2	67 ± 9	59 ± 7	1.07	0.88
UDP	65 ± 4.7	101 ± 23	N.D.	111 ± 3.1	1.57	high
UTP	212 ± 19	639 ± 44	90 ± 3.6	417 ± 32	3.01	4.65
Hypoxanthine	5.2 ± 0.6	8.8 ± 1.3	13 ± 2.4	13 ± 2.1	1.70	0.98
Inosine	1.0 ± 0.1	1.5 ± 0.1	2.1 ± 0.5	1.5 ± 0.4	1.39	0.73
NADH + NAD^+^	351 ± 15	383 ± 15	190 ± 21	140 ± 16	1.09	0.73
NADPH + NADP^+^	127 ± 7	106 ± 7	92 ± 6	62 ± 7	0.84	0.67

^a^Metabolites with statistically significant differences (p < 0.05; Welch’s t-test) between the presence and absence of K^+^ in at least one substrate (glucose or malate) are shaded orange, as are the respective concentration values and ratios.

^b^Mean ± SE of 3–5 experiments. N.D. = below detection limit. Metabolite concentration can also be expressed in μM assuming an intracellular volume of 1 μl/10^9^ cells.

^c^A “high” ratio indicates that the metabolite was not detected in the substrate only condition.

^d^Values in parentheses were determined separately by HPLC/UV quantitation of adenine nucleotides.

### The work function activated by K^+^

The acute and sustained activation of respiration and metabolism concomitant with a very slight increase in cytochrome reduction levels and Δ*p* suggests that one or more energy demanding cellular processes were more active after K^+^ addition. The resulting faster ATP or Δ*p* utilization was balanced by the influx of reducing equivalents into the respiratory chain without proportional changes in redox state, Δ*p*, or high-energy phosphates.

The possible work functions include protein or DNA synthesis in preparation for cell division, or of other high energy storage molecules such as glycogen, lipids, or polyhydroxyalkanoates. Volume regulation is another energy demanding process that could be involved given that cells are not in ionic or osmotic equilibrium with their environment under most conditions. K^+^ depletion occurred in the *P*. *denitrificans* cells used presently as a consequence of the addition of 15% (~1.68 M) glycerol before freezing, or during subsequent thawing. We initially assumed that cells had shrunk in this process and would require energy to move ions across the inner cell membrane in order to recover an optimal intracellular volume and ion composition. We indirectly measured cell volume by monitoring optical density (OD) at a wavelength that would not be affected by changes in cytochrome reduction levels (630 nm). As shown in the representative traces of S3 Fig, addition of 10 mM KCl to K^+^-depleted wild-type cells adapted to respire on malate resulted in a slight and transient swelling (decreased OD) that resumed until ~150 s after KCl addition, when K^+^ intake had been completed. This second phase of swelling coincided with the slightly faster oxygen consumption rate during which no net K^+^ uptake was evident, and stopped approximately when hypoxia was reached. This markedly biphasic swelling pattern was not observed if KCl was added before malate, which resulted in approximately the same maximal respiratory rate accompanied by a monophasic swelling curve. Thus, the observed changes in OD (even if assumed to report only intracellular volume changes) do not correlate with the very rapid activation of respiration that occurs within a few seconds after K^+^ replenishment. The sustained increase in respiration rate after net K^+^ uptake had stopped, also suggested that volume regulation is not the major work function that drives the increased activity of the oxidative phosphorylation pathway in depleted cells upon re-exposure to K^+^. Furthermore, any ion transport process would have to be linked to ATP hydrolysis during the sustained increased in respiration after K^+^ addition as evidenced by its dependence on Complex V activity. We also compared heat generation rates between K^+^-depleted and replenished cells in order to determine if energy was being utilized for synthesis of polymers or other macromolecules, which would result in a somewhat lower heat release rate because of energy being stored in the form of chemical bonds. As shown in S4 Fig for wild-type cells respiring on sodium malate, the relative increase in the rate of heat release upon KCl addition (~3.8) was not statistically significantly different from the increase in respiration (3.1–3.4). Therefore, the same relative amount of energy is being utilized for endergonic reactions in the presence or absence of K^+^.

**Fig 10:**
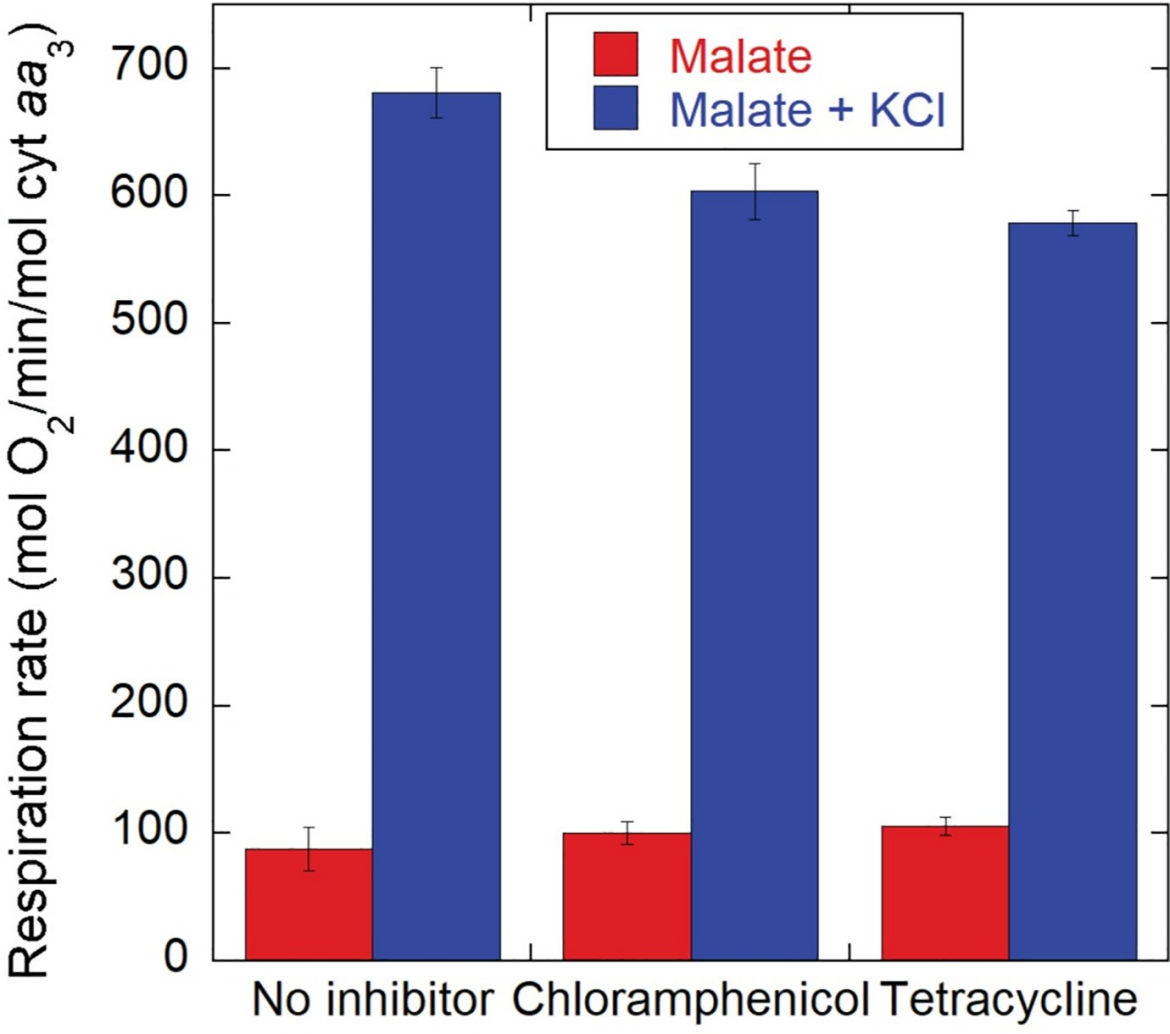
Effect of protein synthesis inhibitors on the activation of respiration by K^+^. Thawed CyoB^-^/CcoN^-^ K^+^-depleted cells (10^9^/ml) grown on malate were incubated with either 4.2 μl of ethanol (solvent effect control), 30 μg/ml of chloramphenicol or 12.5 μg/ml of tetracycline for 9 min before addition of 16.5 mM sodium malate, followed 4 min later by 5 mM KCl. The respiration rates observed during incubation with sodium malate only (red) and after KCl addition were averaged for five independent experiments for each condition. Error bars correspond to S.E. The slight inhibition of the K^+^ activated respiration by chloramphenicol (11%) and tetracycline (15%) relative to the no inhibitor condition was statistically significant (p<0.05; t-test).

Although we observed no net increase in protein concentration in the NH_4_^+^-free medium used for K^+^ replenishment experiments, we considered the possibility that an increased protein synthesis was being driven by the addition of KCl. This increased synthesis could have remained undetected in our protein quantitation determinations simply because of the high protein pool present in the cells or if protein degradation increased. However, as shown in Fig 10, the incubation of K^+^-depleted cells with chloramphenicol or tetracycline at concentrations that largely arrested cell growth by blocking protein synthesis, decreased the K^+^ dependent activation of respiration by no more than 15%. This result indicates that the formation of new peptide bonds consumes very little of the energy made available by the acute activation of oxidative phosphorylation driven by K^+^ replenishment.

## Discussion

The activation of respiration by K^+^ replenishment in *P*. *denitrificans* was characterized as a model for the study of potentially ancient, well-conserved mechanisms of regulation of oxidative phosphorylation by cellular energy demands. *P*. *denitrificans* was selected for these studies because it mimics many of the energy conversion elements and oxidative phosphorylation complexes of mitochondria. A notable finding was the high rate of electron flux of *P*. *denitrificans* cells compared to mitochondria, evident when normalized to cytochrome *aa*_3_ (Complex IV) content to account for the concentration of respiratory complexes present in the membrane. Isolated mitochondria have been reported to approach 700 mol O_2_/min/mol Complex IV at 37°C in porcine heart mitochondria, extrapolating well to intact heart measurements [30]. Similarly, Hoppeler et al. [35] found that the maximum respiratory rate in a variety of skeletal muscle samples was ~5 ml O_2_/min/ml mitochondria, or 667 mol O_2_/min/mol Complex IV assuming 44.63 μmol O_2_/ml gas, 0.33 g of mitochondria protein/ml (37), and 1 mol cytochrome *aa*_3_/mg of mitochondrial protein in skeletal muscle (36). Similar values between 600–700 mol O_2_/min/mol cytochrome *aa*_3_ were also found in *ex vivo* retinal discs and in isolated retinal mitochondria, which are highly oxidative (38). In contrast, the oxygen consumption of *P*. *denitrificans* in growth medium was in the range of 1450–2200 mol O_2_/min/mol Complex IV, depending on the strain and substrate used (see Table 1) at 30°C. Similar rates were found in thawed cells (1400 to 1800 mol O_2_/min; see Fig 1A). Maximal respiratory capacity with uncoupler was as high as 2600–3000 mol O_2_/min in wild-type cells and up to 2600 mol O_2_/min in cells lacking alternative oxidases (see Fig 1B). All these rates determined at 30°C are several-fold higher than the ~700 mol O_2_/min/mol Complex IV in mammalian mitochondria at 37°C even without taking into account the effect of temperature [43].

These observations suggest that the entire respiratory chain of *P*. *denitrificans* is at least 4 times more kinetically efficient in accepting reducing equivalents and generating the Δ*p* necessary for ATP synthesis even when deleting the alternative terminal oxidases not present in mitochondria. *P*. *denitrificans* has a membrane bound cytochrome *c* (*c*552) encoded by the *cycM* gene [44] that has been shown to specifically provide reducing equivalents to Complex IV much more effectively than the soluble cytochrome *c* [45]. This catalytically efficient and less diffusionally limited delivery of reducing equivalents to Complex IV may provide the mechanism for the high reduction rate of oxygen by this enzyme in *P*. *denitrificans*. However, such a high steady state respiratory rate per mol of Complex IV also implies that the reduction of cyt *c*552 must also be equally more rapidly reduced by Complex III. Consequently, ubiquinol reduction by upstream enzymes including dehydrogenases, Complex I, and potentially FADH linked pathways, must also be more rapid than in mitochondria. The mechanisms involved in all these enhancements of catalytic flux efficiency relative to the mitochondria are unknown. It is surprising that this apparent higher kinetic efficiency and its resulting specific power (*i*.*e*., energy conversion rate/unit weight) of the cytochrome chain in *P*. *denitrificans* is not present in mitochondria, since a faster mitochondrial energy conversion system could allow animals to generate more sustained power with the same muscle mass. It has been speculated that the bound cytochrome *c*552 was lost in favor of added control of oxidative phosphorylation [46]. As pointed out earlier, the reduction in flux capacity occurs not only at Complex IV but also in the rest of the respiratory chain, suggesting a significant divergence in the evolutionary pathway that has led to a lower specific power in the “domesticated” mitochondria of eukaryotes when compared to *P*. *denitrificans*.

The activating effect of K^+^ on bacterial respiration has been known since the early work of Krebs *et al*. using *Alcaligenes faecalis* [47] and of Miller and Avi-Dor studying *Pasteurella tularensis* and *Escherichia coli* [48]. The latter group also demonstrated that the activation of respiration by K^+^ in *E*.*coli* depended on the increase of internal K^+^ concentrations and not simply on the inward flux of ions upon addition of external K^+^ [49]. However, little accumulation of K^+^ was observed in *E*. *coli* during the first 10 minutes of incubation with KCl, followed by a phase of rapid increase of intracellular K^+^ during which respiration rate gradually increased, reaching a maximum until 40 min after exposure to KCl. In contrast, in *P*. *denitrificans* we have found that the response of respiration to the reintroduction of KCl in the respiratory medium was very fast, with at least half of its activation preceding any substantial accumulation of K^+^ inside the cells (see Figs 2 and 3) likely reflecting its initial electrogenic influx. Our results on the effect of inhibiting H^+^ flow through Complex V with venturicidin (see Fig 4 and Table 5) confirmed that K^+^ influx results in an early partial depolarization of ΔΨ that increases respiration rate >2-fold. However, respiration is stimulated even further (4-6-fold) after this early phase when ATP synthesis by Complex V is allowed to proceed. Thus, K^+^ influx cannot be solely responsible for the stimulation of respiration by transiently depolarizing the bacterial membrane. The Trk low affinity K^+^ channel found in *P*. *denitrificans* (see Table 2) binds ATP but does not hydrolyze it [50,51], so the electrogenic K^+^ uptake in depleted cells uses ΔΨ as driving force. The following calculation, considering the charges moving across the membrane based on our data, indicates that K^+^ uptake consumes only a fraction of the energy generated by oxidative phosphorylation. Substrate oxidation delivering electrons through Complex I such as glucose and malate result in the translocation of 10 H^+^ to the outside of the plasma membrane per O_2_ consumed. Even in the absence of NH_4_^+^, in which the rate of K^+^ uptake is ~2.4 times higher than the respiration rate (compare Fig 1 and Table 4), only 24% (2–3 K^+^/ 10 H^+^) of the energy generated by the H^+^ pumping complexes of the respiratory chain is being used for K^+^ reentry. Moreover, the activation of respiration persists even after net K^+^ uptake has been completed. Even under the improbable assumption that *P*. *denitrificans* grown at 5 mM KCl could express the high affinity kdp complex (usually expressed at external [K^+^] <100 μM) which hydrolyzes 1 ATP /2 K^+^ transported [52], and assuming ~5 ATP synthesized/O_2_ consumed, only 24% of the ATP generated by oxidative phosphorylation would be dedicated to sustain the initial rate of K^+^ influx. Again, since the stimulation of respiration continued well after the net uptake of K^+^ had ceased, any ATP needed for this high affinity transport cannot be responsible for the sustained increase in respiration after the reintroduction of K^+^.

In earlier work, Erecinska *et al*. [21] were able to partially deplete *P*. *denitrificans* cells of K^+^ by hypotonic shock, which resulted in a ~70% inhibition of respiration when compared to non-depleted or K^+^ replenished cells. These authors also found that the increase in respiratory rates associated with K^+^ replenishment was accompanied by increases in the optical absorbance difference of wavelength pairs that were assumed to report the redox levels of pyridine nucleotides and cytochrome *c*, and by a slight depolarization of ΔΨ concomitant and an increase in ΔpH. However, the absolute ΔΨ values reported were only in the range of -30 to -50 mV, resulting in calculated Δ*p* values insufficient for ATP synthesis, which was incompatible with the high ATP concentration and phosphorylation potential found in K^+^-depleted or replenished conditions. In the present studies, we were able to deplete K^+^ from *P*. *denitrificans* to intracellular concentrations <10 mM to obtain an activation of oxidative phosphorylation by a factor of almost 6 upon K^+^ replenishment (Table 3). Continuous monitoring of ΔΨ and ΔpH using a pH-sensitive GFP expressing strain revealed Δ*p* values >160 mV, fully compatible with ATP synthesis (see Fig 4 and Table 5). Our results demonstrate that the partial depolarization of ΔΨ caused by K^+^ influx is more than compensated by an efflux of H^+^ to generate a ΔpH that results in a slightly higher Δ*p* than in the absence of K^+^. A similar compensatory effect of ΔpH with respect to ΔΨ to maintain an almost constant Δ*p* during K^+^ uptake has been reported previously in *Streptococcus faecalis* and *E*. *coli* [53], indicating that it is widespread among bacteria. In the presence of venturicidin (see Fig 5 and Table 6), a faster electron flow to oxygen was still observed as soon as K^+^ was added, with a ~20 mV depolarization of ΔΨ almost completely compensated by a simultaneous increase in ΔpH. The subsequent partial inhibition of respiration in the presence of the ATP synthesis inhibitor also occurred without net changes in Δ*p* as a ~9 mV ΔΨ repolarization was compensated by an equivalent decrease in ΔpH. Therefore, the respiratory rate appears to be more dependent on the electrogenic flux of ions and, at least partially, on the ATP synthesis activity of Complex V than on the driving force (Δ*p*). The degree of inhibition exerted by venturicidin on respiration (~50%) is similar to that reported in inverted vesicles of *P*. *denitrificans* [34], even though it completely inhibits the ATP synthase activity of Complex V [54]. Thus, the respiratory control exerted by the ATP concentration in this bacterium is lower than what is observed in isolated mitochondrial preparations, in which Complex V inhibitors such as oligomycin inhibits respiration almost completely, especially if a high degree of coupling is maintained.

We have characterized the effects of K^+^ on cytochrome redox levels using integrative sphere spectroscopy and multiwavelength spectral fitting (see Figs 7–9), which we have previously applied to the study of electron flux in isolated mammalian mitochondria [35,36] and intact hearts [55]. We also attempted the quantitation of changes in NADH concentration by fluorescence, but the spectral shape and the diffuse distribution observed by high resolution microscopy within the bacterial cells cast doubt on the molecular origin of the detected emission, which in mitochondria is limited to NADH binding to Complex I [56]. Nevertheless, the small relative change we observed in the cytochrome redox level (see Fig 7) upon a several fold activation of respiration is reminiscent of the reported changes in isolated mitochondria upon calcium activation of oxidative phosphorylation [36,57] that are evidence of a parallel activation of the Δ*p*-generating (dehydrogenases and respiratory complexes) and Δ*p*-consuming (ATP synthase) components of the pathway. The fact that this homeostatic regulation is present in *P*. *denitrificans* highlights the ancient and highly conserved nature of the parallel activation mechanism involved, even if its triggering agent is different. However, the dominant contribution to the absorbance changes observed upon activation of respiration by KCl addition is a reduction of cytochrome *b* species (*b*558 and *b*_H_; see Figs 8D and 9D). This was not as prominent in isolated mitochondria upon stimulation by addition of ADP, even in the presence of calcium, which renders *b*_H_ reduction slightly more evident [35,36,57]. Nevertheless, mitochondrial difference spectra show an oxidation of *b*_L_ because of ΔΨ depolarization and increases in cytochrome *c* and *a*607, which are also detected in *P*. *denitrificans* (see Fig 8B and 8D).

A very different spectral pattern was observed in the absence of K^+^. Addition of substrate caused a slight decrease in the Complex IV *a*607 species (see Fig 8A and 8B) in spite of an increase in respiration relative to the absence of substrate (see Table 4). The absorbance of *a*607 increases linearly over a wide range of respiration rates and ΔΨ values in isolated mitochondria [35,36], and also by addition of uncoupler in the absence of K^+^ in *P*. *denitrificans* (see Fig 9A and 9B). Substrate addition in the absence of external K^+^ results in a hyperpolarized (as shown by ΔΨ in Fig 5 and the dominant *b*_L_ contribution in Fig 8B) but low flux condition in which electrons are not able to accumulate within Complex IV.

The increased reduction level of *b*-type cytochromes upon re-exposure to K^+^ is consistent with our metabolomic data that indicate a faster delivery of reducing equivalents from glycolysis and the TCA cycle (see Table 6). A direct activation by K^+^ ions is known for several enzymes (from bacteria or other organisms) involved in glycolysis, the TCA cycle, and gluconeogenesis such as phosphofructokinase [58], aldolase [59], pyruvate kinase [60], succinyl-CoA ligase [61], pyruvate carboxylase [62], and phosphoenolpyruvate carboxykinase [63]. The marked increase in citrate in contrast with the apparent reduction of the acetyl-CoA pool when K^+^ was replenished is consistent with increased activity of citrate synthase. The large accumulation (at least 6-fold) of 2-oxoglutarate would favor the mobilization of nitrogen into the synthesis of other amino acids and bases by favoring its transamination into glutamate, which also increased in concentration (see Table 7). Glutamate was high even before addition of K^+^, increased by 2-fold in the presence of glucose and stayed constant with malate as substrate, implying that its γ-amino group was being rapidly removed by glutamate dehydrogenase and other enzymes to form other amino acids such as proline, which doubled in the presence of K^+^. The sharp decrease observed in glutamine, lysine and (to a lesser extent) in arginine indicates that side chain amino groups are also removed upon activation of metabolism by K^+^. Even though glutamine synthetase is known to be activated by K^+^ [64,65], it is also reported to act only in the synthetic direction, with very low activity in the direction of ATP synthesis coupled to removal of the γ-amino group [65]. Thus, the decrease in glutamine is probably due to the activity of other enzymes that in bacteria can utilize the amide group of glutamine, including glutamate synthase, glutamine-fructose-6-phosphate transaminase, and carbamoyl phosphate synthase [66]. A higher activity of this last enzyme after addition of K^+^ is supported by the observed ~2-fold increase in citrulline concentrations.

Amino groups from glutamine are needed for the synthesis of purines. Therefore, the increase in the pools of adenine and guanine nucleotides (see Table 8) is also consistent with the pronounced decrease in glutamine levels in the presence of K^+^. Among pyrimidines, uridine levels were also higher, and since their synthesis starts with carbamoyl phosphate, glutamine could also be consumed by this synthetic pathway, unless other sources of nucleotides, such as ribosomal RNA breakdown [42] are sufficient. Whatever the source may be, the increase in the pool of almost all nucleotides in the presence of K^+^ suggests that the cell is preparing to synthesize DNA even in the absence of NH_4_^+^. To do so, energy needs to be invested in phosphorylating the resulting NMPs generated by either *de novo* nucleotide synthesis or RNA hydrolysis. However, the resulting higher concentrations of NTPs and ATP/ADP ratios achieved in the presence of K^+^ clearly did not exert any inhibition on the >4-fold K^+^-dependent activation of respiration, highlighting a different and as yet unidentified control mechanism of oxidative phosphorylation. This mechanism involves the activation of ATP turnover, as evidenced by the venturicidin-sensitive sustained depolarization of ΔΨ observed after K^+^ addition (see Tables 4 and 5), which likely drives the sustained increase in respiration with K^+^ reintroduction.

Despite the increase in the content of high energy phosphate bonds in nucleotides, heat generation relative to respiration rate did not change (see S4 Fig), which would occur if a significant amount of energy was being converted into chemical work (instead of being lost as heat) by catalyzing a higher rate of endergonic reactions. Dividing the heat generation rate by that of respiration, our results indicate that ~67 kcal were released per mol of O_2_ consumed in the absence of K^+^, very close to the slightly less than 80 kcal/O_2_ generated when K^+^ was present. Consistent with this observation, protein synthesis inhibitors exerted only a small effect on K^+^ activated respiration (see Fig 10), indicating that peptide bond formation is not a dominant work function stimulated in the K^+^ replenished cells, likely because of the lack of a sufficient nitrogen source. Regulation of cell volume and turgor pressure have been proposed to be a major role of K^+^ in bacteria [27], and have long been known to be dependent on respiration [67–69], including in *P*. *denitrificans* [70]. The active transport of ions is one of the most energetically costly cellular processes, and even passive ion fluxes can result in higher heat generation than enzymatic reactions. However, our kinetic results on volume changes (see S3 Fig) failed to reveal a clear association between the almost immediate activation of respiration by K^+^ and the delayed swelling events indicative of the entry of ions that occurred even after K^+^ uptake had reached completion. Future detailed studies could dissect whether the exchange of different ions without discernible optical effects on cell volume is the elusive work function that is acutely activating oxidative phosphorylation upon K^+^ replenishment in these bacteria.

In summary, *P*. *denitrificans* has a very high respiratory capacity relative to the content of oxidative phosphorylation complexes indicating a more kinetically efficient electron transfer system with higher specific power compared with mammalian systems. K^+^ addition induces a rapid initial activation of respiration due to its electrogenic influx, followed by a sustained simultaneous activation of substrate delivery and energy utilization dependent on ATP recycling. This parallel activation that includes oxidative phosphorylation occurs with little change in driving force, similar to the energy homeostasis observed in mammalian tissues. These observations imply that the mechanism regulating oxidative phosphorylation during large and variable increases in energy demand is conserved between *P*. *denitrificans* and mammalian *in situ* mitochondria. We expect that future studies with this bacterial system will characterize the mechanism for the coordinated regulation of energy generation and utilization pathways that may be in play in the regulation of oxidative phosphorylation in mammalian tissues.

## Methods

### Cell strains and growth conditions

A 14 L New Brunswick^TM^ BioFlo 115 fermenter with a working volume of 9.5 L was used for large-scale growth of *P*. *denitrificans* strain 1222, obtained from Prof. Stephen Spiro, Department of Biological Sciences, University of Texas at Dallas. The fermenter was filled with growth medium containing 50 mM Na_2_HPO_4_, 75 mM NH_4_Cl, 11.5 mM Na_2_SO_4_, 1.1 mM citric acid, 5 mM KCl, 4 μg/L biotin, and either 11.1 mM glucose or 16.5 mM malic acid. 10 ml/L trace metal solution (Ludwig 1986) and 1.25 ml/L of 1 M MgCl_2_ were added to the fermenter before inoculation, and pH was adjusted to 6.9. The fermenter was inoculated with a sample of *P*. *denitrificans* cells pre-adapted to the appropriate carbon source to reach an initial concentration of cells of 10^5^ cells/mL (OD_600_ = 0.0001) and started with an initial agitation of 300 rpm and an airflow of 2 L/min. Temperature was maintained at 30°C, and dissolved oxygen was kept at 80% relative to room air by increasing agitation during cell growth. We determined that this level of oxygen was necessary to maximize the concentration of cytochrome *aa*_3_ (Complex IV) in the cells. pH was maintained at 6.9 in the case of malate growth medium, and allowed to vary in glucose medium, reaching a value of 6.7 at the end of the culture period. BioCommand Batch Control software from New Brunswick^TM^ was used for online data collection. A 1313 fermentation monitor with BZ6009 requisition software (California Analytical Instrument) was used to measure gas O_2_ and CO_2_ concentration. Cells were harvested at ~1.5x10^9^ cells/mL (OD_600_ = ~1.5) using a Sharple continuous centrifuge at a flow rate of 250 mL/min. Cell pellets were re-suspended with the above medium plus 15% glycerol in the cold room to a density of OD_600_ = 60, and aliquots of 5 ml were stored in 15 ml tubes at -80°C.

#### Generation of CyoB^-^ and CyoB^-^/CcoN^-^ strains

In our hands, CRISPR/Cas9 was ineffective in *P*. *denitrificans*. We thus utilized the following procedure to generate deletions in the alternative oxidases. Genomic DNA was isolated by growing 1 ml of wild-type cells overnight in the growth medium described above, and harvesting the cells by centrifugation. The cell pellet was resuspended in 0.5 ml of cell lysis buffer containing proteinase K (100 μg/ml, Promega), and incubated at 50°C for 2h. 500 ul of isopropanol was added to precipitate genomic DNA. Genomic DNA was washed with 80% ethanol three times, and dissolved in 300 ul TE buffer (Invitrogen, 8019005). A 4.2 kbp PCR product containing the *cyoB* gene (2,007 bp long) in the middle was made by using primers with sequence 5’-gaggaatccgatgcgatccagattcg-3’ and 5’-tttcgaagccgccggccatgaccaggcc-3’. The *cyoB* gene (2,007 bp long) was then replaced with the kanamycin gene in the pARO181 vector (mobilizable suicide vector, ATCC 77125). Donor cells, *E*. *coli* S17-1 (ATCC 47055), were transformed with the gene replacement cassette, and conjugated with *P*. *denitrificans* wild-type cells as receiver cells by mixing at a 1:4 ratio, and incubated at 30°C for 4h without shaking. The conjugated cells were shaker-cultured in kanamycin (50 ug/ml) containing medium supplemented with succinate overnight at 30°C. Cells were plated on a kanamycin-containing agar plate (50 ug/ml). Colonies were screened by colony PCR for gene replacement cassette integration into wild-type cell chromosomal DNA by single-crossover (SCO) cells. For screening SCO cells, a primer set was used: the forward primer 5’-gat ccg gct gcg gat cgg gcg cgg aac ttc-3’ and the reverse primer 5’-ctc aag ctc cca gta ttg gcg cat gg-3’. Two PCR products could be generated from SCO cells: 2.1 kb from wild-type and 1.6 kb from mutant. If only one PCR product was detected, cells were either wild-type or mutant cells. SCO cells were serially sub-cultured to increase the number of the double-crossover (DCO) cells in which a gene is replaced with the kanamycin gene. For serial passage culture, 1 ml was transferred from a 5 ml overnight culture at 37°C of SCO cells to a 5 ml fresh medium culture in the absence of kanamycin every day for 21 days. Then cells were plated on agar plates for colony PCR. Genomic DNA was prepared from DCO cells for DNA sequencing. To generate the *cyoB^-^/ccoN^-^* strain, *cyoB* gene deleted mutant cells were mated with S17-1 cells transformed with the suicide vector pARO181 containing the *ccoN*/streptomycin gene replacement cassette. For DCO cells the same strategy was used as above. The double *cyoB/ccoN* gene replacement was confirmed by both PCR and DNA sequencing. These newly generated strains will be deposited at the ATCC (American Type Culture Collection), along with the GFP expressing strain described next.

### Expression of pH-sensitive GFP

The Tn5 promoter fragment (from pRVS1) was fused to three copies of the pH-sensitive ratiometric *gfp* open reading frame (from pUV15-pHGFP, Addgene Plasmid# 70045) [71–73]. The *gfp* gene (3 copies) was integrated into genomic DNA using a suicide vector (named pRVScycMAS3) in which the fused *gfp* replaced a copy of the *cycM* gene that encodes the membrane-bound cytochrome *c* (*c*552). *P*. *denitrificans* cells were transformed with pRVScycMAS3 by conjugation as described before [74]. The vector was integrated into the region adjacent to the *cycM* gene in the chromosomal DNA by single crossover. In the presence of streptomycin as selection marker, *P*. *denitrificans* cells remained with the integrated vector. pH-sensitive GFP expression was confirmed by Western blot and fluorescence microscopy. The expression of this protein did not affect *cycM* gene expression. Incorporation of 3 copies was necessary to obtain sufficient fluorescence signal for intracellular pH measurements.

### Spectroscopic quantitation of cytochrome *aa*_3_

A 5ml aliquot of cells was thawed at 4°C using a Rotamix rotator (model RKVSD, Appropriate Technical Resources, Laurel, MD) set to a speed of 15 rpm for 35 min, or until all the sample was thawed. The cell sample was diluted to 40 ml with growth medium and centrifuged at 7700g for 8 min to remove the glycerol. The cell pellet was resuspended in 2.2 ml of growth medium, and cells were disrupted by passing the suspension twice through a One Shot cell disruptor (Constant Systems, Ltd, Daventry, UK) set at a pressure of 40,000 psi. 0.2 ml of the disrupted cell sample was added to 0.7 ml of 5 mM K_2_HPO_4_/KH_2_PO_4_ buffer (pH 7.0) and 0.1 ml of 10% n-β-D-dodecyl maltoside (DM) was added to solubilize membrane-bound cytochromes. After vortexing for 40 sec, the sample was centrifuged for 2 min at 16,000g, and 0.95 ml of the supernatant was recovered and transferred to a 1 ml cuvette to record the oxidized spectrum in the wavelength range of 500 to 650 nm in a commercial spectrophotometer (Shimadzu 2700 UV-Vis; Shimadzu Corp., Kyoto, Japan). Addition of 2 ml of 0.1 M K_3_[Fe(CN_6_)] to ensure full oxidation of cytochromes was needed given that a partial reduction of the cytochromes likely driven by endogenous substrates present in the sample was observed. A few grains of sodium hydrosulfite were added to fully reduce the sample. The oxidized spectrum was subtracted from the reduced spectrum, and the absorbance difference at 607 nm was calculated after manually tracing a baseline from 575 and 630 nm. An extinction coefficient of 12 mM^−1^cm^−1^ was used to obtain cytochrome *aa*_3_ concentration, as we have reported for mammalian heart mitochondrial Complex IV [75], which was confirmed by copper quantitation by atomic absorption spectroscopy of Complex IV isolated from *P*. *denitrificans* DM-solubilized membranes by anionic exchange chromatography using a procedure originally reported for Complex III purification [76] with modifications. Disrupted cells (corresponding to 200 nmol of cytochrome *aa*_3_) were thawed and diluted to 10 nmol cytochrome *aa*_3_/ml in ice cold buffer A (50 mM Tris, 1 mM MgSO_4_, adjusted to pH 8.45 at 4°C) before dropwise addition of 10% w/v n-β-D-dodecyl maltoside (DM) to a final detergent concentration of 1% on ice with gentle mixing. This extract was centrifuged at 40,000g for 40 min at 4°C and the supernatant was applied to a 2.5x20 cm glass column (Bio Rad) previously packed with DEAE Sepharose Fast Flow anion exchange resin (GE Healthcare) equilibrated with 5 column volumes buffer A containing 0.02% w/v DM. After washing with 2 column volumes of buffer A with 0.02% DM, elution of cytochrome complexes was achieved by applying a linear 0–400 mM NaCl gradient in the same buffer, and collecting the eluate in 4 ml fractions. Green fractions containing Complex IV eluted close to the middle of the gradient (~180 mM NaCl) before the reddish fractions containing Complex III. The pooled Complex IV containing fractions were concentrated to 20–30 nmol cytochrome *aa*_3_/ml in an Amicon Ultra-15 centrifugal filter (Millipore) with a 10 kDa membrane cutoff. Sodium ascorbate- or hyposulfite-reduced minus air- or ferricyanide-oxidized spectra showed no contamination by *b* or *c*-type cytochromes. Validation of the extinction coefficient for the fully reduced cytochrome *aa*_3_ was done by quantifying copper in this spectroscopically pure Complex IV preparation by atomic absorption spectroscopy (AAS) using a PinAAcle 900Z instrument (Perkin Elmer) equipped with a graphite furnace and a hollow cathode lamp for copper and calibrated with a 10–320 μg/l copper standard solution. A Complex IV sample of known absorbance was digested in 6% HNO_3_ for 20 min in boiling water and centrifuged at 16,000g for 10 min; the supernatant was then placed in the AAS instrument for copper quantitation. After subtracting for the background AAS absorbance using an equivalent volume of buffer C + 6% HNO_3_, the copper amount was divided by the optical absorbance considering 3 Cu/cytochrome *aa*_3_ yielding an extinction coefficient of 12.5 ± 0.2 mM^-1^ cm^-1^ (n = 3), almost identical to the value of 12 mM^-1^ cm^-1^ reported previously for mitochondrial Complex IV [75].

#### K^+^ depletion of cells

After thawing a 5 ml aliquot of frozen cells as described above, the cell suspension was taken to a volume of 40 ml with ice-cold depletion buffer (50 mM Na_2_HPO_4_, 11.5 mM Na_2_SO_4_, 85 mM NaCl, 1.25 mM MgCl_2_, adjusted to pH 7.0) in a centrifuge tube and the cells were spun down at 7700g for 8 min at 4°C. Enough depletion buffer (2–3 ml) to submerge the cell pellet was added, and the pellet was resuspended using a transfer pipette. The volume was taken to 40 ml by adding more ice-cold depletion buffer, and the previous centrifugation steps were repeated three more times. The final cell pellet was resuspended after adding 1 ml of depletion buffer. Cell density in this final suspension was calculated by adding 5 μl of cells to 995 μl of depletion buffer in a 1 ml cuvette and recording the absorbance at 630 nm, assuming 1 absorbance unit = 10^9^ cells/ml. Intracellular K^+^ concentration before and after depletion, or after replenishment, was determined by spinning down a known number of cells (10–50 x 10^9^) at 13,000g for 10 min, carefully removing all the supernatant, and resuspending in 380 μl of depletion buffer. Cells were lysed by adding 20 μl of 10% trifluoracetic acid while vortexing, incubated for 2 min at room temperature, and neutralized with 4 μl of 5 M NaOH. After adding 100 μl of 10% DM, the sample was centrifuged at 16,000g for 1 min to sediment the insoluble material, which was found to have a negligible amount of K^+^ by atomic absorption spectroscopy. The supernatant (400–450 ml) was collected and added to a cylindrical flat bottom glass vessel containing 4.6 ml of depletion buffer in which a K^+^-specific electrode (MI-442, Microelectrodes, Inc.) and a flexible reference electrode (MI-402, Microelectrodes, Inc.) connected to a pH/Millivolt meter (PHM 93, Radiometer Copenhagen). After adding the cell lysate supernatant stepwise and recording the resulting voltages, 0.1 mM and 0.9 mM KCl were added to record the corresponding voltages (V) of x + 0.1 mM and x + 1 mM, where x is the [K^+^] in the chamber contributed by the lysate. Calibration was performed separately by adding known concentrations of KCl in the range 0.1–10 mM to 5 ml of depletion buffer to obtain the slope value (m), which was typically of 55–57 mV per 10-fold change in KCl concentration. The recorded values were used to solve the following equation for x:

$$\frac{x+1\;mM}{x+0.1\;mM}=10^{\left(\frac{V_{\left(x+0.1\;mM\right)}-V_{\left(x+1\;mM\right)}}{m}\right)}$$

For estimating the intracellular [K^+^] from x, an intracellular volume of 0.9 and 0.83 μl/10^9^ cells was assumed for glucose- and malate-grown cells, respectively. These volume numbers were calculated after determining a dry weight of 0.28 mg/10^9^ glucose-grown cells and 0.26 mg/10^9^ malate-grown cells, and assuming an intracellular volume of 3.2 μl/mg dry weight. Typical intracellular [K^+^] values after depletion were in the 6–12 mM range, whereas in non-depleted or K^+^ replenished cells values were 86–90 mM.

### Simultaneous measurement of O_2_ consumption, K^+^ or tetraphenylphosphonium (TPP^+^) uptake, and NADH or intracellular pH-sensitive GFP fluorescence

Custom made flat-bottom cylindrical glass vessels of 20 mm outer diameter (1.6 mm wall thickness, 16.4 mm inner diameter) x 60 mm height coated on the outside with black paint and fitted on top with a black plastic stopper with five holes drilled through the top to accommodate four electrodes/light fibers and one Hamilton syringe (for reagent addition) were used as reaction chambers. A vessel containing a total volume of 4.2 ml of depletion buffer (including cells) was fitted inside a hole of a metal block heater (Multi-Blok 2053, Lab-Line) set to 30°C and a magnetic stir bar controlled with a Telemodul electronic stirrer was placed inside the vessel. For O_2_ consumption measurements, an Ocean Optics RE-BIFBORO-2 bifurcated probe assembly with a RedEye oxygen sensing patch (RE-FOS) attached to the tip and connected to a NeoFox-GT fluorometer was fitted through one of the stopper holes of the reaction vessel. For conversion of electrode voltage to O_2_ concentration, room air oxygen concentration in solution was assumed to be 0.23 mM. For measuring K^+^ or TPP^+^ concentration in the chamber, a K^+^-specific electrode (MI-442, Microelectrodes, Inc.) or an ion-selective electrode (KWIKTPP-2, World Precision Instruments), respectively, together a flexible reference electrode (MI-402, Microelectrodes, Inc.) connected to a pH/Millivolt meter (PH-1, Microelectrodes, Inc.). For measuring NADH or intracellular pH-sensitive GFP fluorescence, a high-power fiber-coupled LED light source of 365nm (UV FCS-0365-000, Mightex) or high-power fiber coupled 410 and 470 nm led lights (Mightex) gated and controlled by a BioLED driver (Mightex), respectively, were used as light sources. In both cases, a Mightex Sirius Universal LED Controller (SLC-SA/SV/AA/AV Series) coupled to an Ocean Optics (QE65000) Scientific-grade Spectrometer were used to collect emission spectra. Data was visualized and collected using a custom written LabView program. [K^+^] in the solution was calculated by calibrating in the absence of cells with known amounts of KCl in the range of 0.1–10 mM and fitting the corresponding electrode voltage readings to log [K^+^] by linear regression to obtain a slope value, which was typically in the range of 54–55 mV per 10-fold change in KCl concentration.

We found that using 2 μM of TPP^+^ chloride resulted in better uptakes (>80% at the highest ΔΨ) than the use of butyl-triphenylphosphonium (butyl-TPP^+^) bromide, which even at the suggested concentrations of 10 μM [22] was not as permeable as TPP^+^. We also noticed that butyl-TPP^+^ generated a precipitate in the electrode filling solution, apparently by reaction with the silver wire, which rendered the electrode nonfunctional after a few days. The electrode was filled with a solution of 1 mM TPP^+^ and 100 mM NaCl. [TPP^+^] outside the cells was calculated after fitting the corresponding electrode voltage readings in the absence of cells to log [TPP^+^] in the range of 0.1–19.2 μM by linear regression to obtain a slope value, which was typically in the range of 58–60 mV per 10-fold change in [TPP^+^], and assuming that the missing TPP^+^ was inside the cells at a concentration calculated by considering the intracellular volume as 0.9 and 0.83 μl/10^9^ for glucose- and malate-grown cells, respectively. The difference in concentration between the outside and the inside of the cells was used to quantify the membrane potential (ΔΨ) with the Nernst equation after subtracting the bound TPP^+^ as determined by adding the uncoupler 2,6-di-tert-butyl-4-nitrophenol (DBNP) at the end of each experiment. We found a small additional ΔΨ depolarization of ~25 mV after oxygen was apparently depleted when 1 mM sodium cyanide was added to fully block respiration, indicating that a slow oxygen consumption was still occurring (S5 Fig). Furthermore, the oxygen signal slowly drifted upward in the presence of NaCN, consistent with a low oxygen leak into the chamber through the port hole used for reagent addition. This leak was quantified to be of 2.4 μM O_2_/min.

pH was determined using the wild-type strain grown on malate integrated with 3 copies of the pH-sensitive GFP (see above), which is differentially excitable depending on pH at 410 (alkaline) and 470 nm (acidic) wavelengths with a 510 nm peak emission wavelength. Each experiment was collected in either GFP or wild-type background with either 410 or 470 nm excitation. For each condition (*i*.*e*., no substrate, sodium malate adjusted to pH 7.0, KCl addition), 30 points were averaged and the background fluorescence from cells not expressing GFP was subtracted after acquisition. After hypoxia, 3 μM nigericin followed by 0.5 μM DBNP were added to abolish ΔpH and ΔΨ, respectively. The resulting emission between 510 and 520 nm when exciting at 410 nm relative divided by that obtained at an excitation of 470 nm was averaged to determine intracellular pH at each condition (*i*.*e*., substrate, KCl) based on a pH calibration curve generated in the same cells suspended in depletion buffers with a pH in the range of 6–8 in the presence of nigericin to equalize internal and external pH. The ΔpH between conditions was determined and converted to mV assuming 1 pH unit = 59.16 mV according to the Nernst equation. It should be noted that the signal provided by the pH-sensitive GFP probe inside the cells provides instantaneous monitoring of intracellular pH, whereas monitoring of ΔΨ using the ion selective electrode that monitors extracellular TPP^+^ concentrations is delayed by the several seconds due to the diffusion of TPP^+^ through the cell membranes and the response time of the electrode.

### Quantification of cytochrome redox state in intact cells

The electron distribution within and between respiratory complexes in *P*. *denitrificans* cells was determined using integrating sphere spectroscopy as described before for mitochondrial suspensions [35]. This technique allows the accurate determination of absorbance spectra in highly turbid suspensions of isolated organelles or intact cells by minimizing the effects of light scattering. In this setup, light is detected after a high number of reflection events inside a sphere coated with a highly reflective white material, maximizing the probability of all photons passing through the sample. Cells were added to a final volume of 4.5–5 ml of depletion buffer in a cylindrical glass vessel with magnetic stirring, and either substrates or KCl were added with a Hamilton syringe through a port hole above the vessel. Oxygen was provided by blowing room air that was heated to 30°C using a humidification system (Neo-Pod “T”, Westmed, Inc.). Spectra were collected at a rate of 2 Hz and analyzed in the range of 530–630 nm and fitted as previously reported [35] using as references individual cytochrome species separated by anionic exchange chromatography of *P*. *denitrificans* DM-solubilized membranes. Full reduction of cytochromes was achieved by stopping air flow after addition of substrate and KCl.

### Volume regulation measurements

Cells were added to 2 ml of experimental buffer in a 3 ml plastic cuvette and placed in a commercial spectrophotometer (Shimadzu 2700 UV-Vis; Shimadzu Corp., Kyoto, Japan) with magnetic stirring and water jacketed temperature control set to 30°C. Absorbance was measured at 630 nm, which was determined to be a wavelength at which contribution from changes in the redox state of cytochromes is minimized, at a rate of 1 data point/sec. It was determined by addition of hypertonic concentrations of NaCl that absorbance at this wavelength is inversely proportional to cell volume.

### Determination of heat generation by microcalorimetry

Bacterial cells were thawed and washed, depleting them of substrate and K^+^ using the depletion buffer and procedure described above. Cells were diluted to 2.5x10^9^ cells/ml (2.5 OD) in 100 μl ice-cold growth medium or depletion buffer containing (1) no substrate, no KCl; (2) 16.49 mM sodium malate only; (3) 16.49 mM sodium malate and 5 mM KCl. The automated rotating syringe of an iTC200 (Malvern) microcalorimeter was loaded with a maximum volume of ~43 μl of each sample. The syringe was then transferred to the chamber with buffer until ready for injection (~10 min). 500 μl of distilled water was added to the chamber and aspirated out for cleaning followed by a rinse with the same volume of depletion buffer. 450 μl of depletion buffer (with or without sodium malate and/or K^+^) or growth medium was added to the clean chamber. The reference power was set to 10 μCal/s for baseline. The initial delay was set to 120 s to report a buffer only baseline before injection. The stirring speed of the syringe was set to 750 rpm and the collection time was set to 2000 s. After the start of data collection, the instrument requires ~10 min to calibrate and warm to 30°C. Once calibrated, a baseline with depletion buffer only was collected for 120 s. 38 μl of cells from each incubation condition was injected from the automated syringe into the microcalorimeter chamber and the rate of heat generation was collected for 2000 s.

### Effect of protein synthesis inhibitors on growth and respiration

A frozen stock of CyoB^-^/CcoN^-^ cells grown in malate was inoculated into 20 ml growth medium in a 250 ml plastic shake flask and incubated at 30°C overnight in New Brunswick Innova 4230 Refrigerated Incubator Shaker set to 220 rpm. The next day, an aliquot of 0.67 ml was added to 20 ml of fresh growth medium contained three separate 250 ml plastic shake flasks. After 90 min, the OD at 600 nm was checked and chloramphenicol (final concentration of 30 μg/ml) or tetracycline (12.5 μg /ml) was added to one of the flasks, and incubated at 220 rpm and 30°C. The OD was measured 10, 20, 30 and 120 min later. The increase in OD in the control flask was inhibited by 85–90% in the flasks containing either chloramphenicol or tetracycline. Similar results were obtained in separate incubations with higher concentrations of chloramphenicol (100 or 200 μg/ml) or tetracycline (25 or 50 μg/ml). Therefore, the lower concentrations were chosen for respiration experiments. For these, cells were thawed and washed using depletion buffer and respiration and K^+^ uptake measurements were performed as explained above. Cells were incubated at a density of 10^9^/ml at 30°C in the presence of either 4.2 μl of ethanol (as solvent control), 30 μg/ml of chloramphenicol, or 12.5 μg/ml of tetracycline for 9 min. Then, 16.5 mM of sodium malate was added followed by the addition of 5 mM KCl 4 min later.

### Proteomics

Glucose and malate grown cells from both wild-type and CyoB^-^/CcoN^-^ strains were disrupted by passing the suspension twice through a One Shot cell disruptor (Constant Systems, Ltd, Daventry, UK) as described above and protein was precipitated with 6 volumes of cold acetone overnight. After sedimentation by centrifugation at 10,000g for 10 min at 4°C, protein was resuspended in 100 μl of lysis buffer containing 6 M urea, 2 M thiourea and quantified using the Pierce detergent compatible Bradford assay kit (Thermo Fisher Scientific). A volume corresponding to 100 μg of protein for each strain and substrate condition was transferred to 5 separate tubes, as well as two normalization loading control tubes in which 5 μg of each replicate were pooled, were taken to 100 μl by adding the necessary volume of lysis buffer. Each resulting sample was reduced by mixing with 5 μl of the 200 mM DTT and incubating at room temperature for 1 hour, and then alkylated by adding 5 μl of 375 mM iodoacetamide and incubating for 30 minutes protected from light at room temperature. Urea was diluted to 1 M by adding 500 μl of 100 mM triethylammonium bicarbonate (TEAB). Each sample was proteolyzed by adding 10 μl of 1.25 μg/μl sequencing grade modified trypsin (Promega) dissolved in 100 mM TEAB and incubated overnight at 37°C. Each wild-type and CyoB^-^/CcoN^-^ replicate and loading control was labeled with a different TMT label reagent from an 11-plex set (Thermo Fisher Scientific) by adding the contents of each label tube after dissolving with 41 μl of acetonitrile. The reaction was allowed to proceed for 1 hour at room temperature, and was quenched by adding 8 μl of 5% hydroxylamine to each sample and incubating for 15 minutes. All ten samples from each strain (plus a normalization loading control) were combined in a new microcentrifuge tube, which was dried under vacuum until all acetonitrile was removed. The combined samples were desalted using an Oasis HLB column (Waters) and dried under vacuum. High pH reversed-phase liquid chromatography and mass spectrometry data collection were performed as described in detail elsewhere [77] Raw data files were processed using Proteome Discoverer (v2.4, Thermo Fisher Scientific), using the Sequest HT (Thermo Fisher Scientific) search algorithms. All the peak lists were searched against the non-redundant protein Reference Sequence Database (RefSeq) with *P*. *denitrificans* taxonomy (5,012 sequences as of December 2019) and concatenated with reversed copies of all sequences. The following search parameters were set as static modifications: carbamidomethylation of cysteine, TMT 11-plex modification of lysine and peptide N-terminus and a variable modification of methionine oxidation. For SPS-MS^3^ the precursor and fragment ion tolerances of 10 ppm and 0.5 Da were applied, respectively. Up to two-missed tryptic cleavages were permitted. Percolator (v3.02.1, University of Washington) algorithm was used to calculate the false discovery rate (FDR) of peptide spectrum matches, set to q-value 0.05 [78–81]. TMT 11-plex quantification was also performed by Proteome Discoverer v.2.4 by calculating the sum of centroided ions within 20 ppm window around the expected m/z for each of the 11 TMT reporter ions. Spectra with at least 50% of SPS masses matching to the identified peptide were considered as quantifiable PSMs. Quantification was performed at the MS^3^ level where the median of all quantifiable PSMs for each protein group was used for protein ratios.

### Metabolomics

Wild-type cells grown in glucose or malate were thawed and depleted of K^+^ in substrate-free buffer as described above, and ~8 x 10^9^ cells were transferred to the custom made flat-bottom cylindrical glass vessels used for respiration studies (see above) containing 4.2 ml of depletion buffer supplemented with 11.1 mM glucose or 16.49 mM sodium malate with or without 10 mM KCl for 20 min in a 12620–946 Incubating Orbital Shaker (VWR) set to 30°C and 400rpm. Five replicates were done for each condition. The contents of each vessel were transferred to 40 ml of ice-cold depletion buffer and centrifuged at 10,500g for 4 min at 4°C. 500 uL of 100% MeOH stored at -80°C was used to resuspend the cells, and then 1800 uL of a 50% MeOH/water mixture was added. A mixture of metabolite internal standards provided by Human Metabolome Technologies (HMT, Boston) was present in the MeOH used at a concentration of 5 μM. After cell breakage using the One Shot cell disruptor as described above, 1600 uL of each cell suspension was mixed with 1600 μL of chloroform and 640 μL MilliQ water. 1500 μL of the aqueous phase was collected and filtered using Amicon 0.5 ml Ultra centrifugal filters with a 3kDa MW cutoff (Millipore Sigma). The filtrates for each sample were split into 3 aliquots of 500 μL each, dried and stored at -80°C. One aliquot was used to quantify AMP, ADP, ATP, and GTP by HPLC/UV as previously published [82]. Another aliquot was analyzed by Human Metabolome Technologies (HMT) in Tsuruoka, Japan. Samples were resuspended with 25 μL or 250 μL of ultrapure water for cationic and anionic CE-TOFMS measurement, respectively, using the Agilent system (Agilent Technologies Inc). In both cases a fused silica capillary with internal diameter of 50 μm x 80 cm and a pressure injection of 50 mbar was used. For detection of cationic metabolites, the injection time was 10 s with a positive 27kV CE voltage, ESI positive MS ionization and a MS capillary voltage of 4 kV. For anionic metabolite detection, the injection time was 25 s with an ESI negative MS ionization and MS capillary voltage of 3.5 kV. In both cases the MS scan range was set to 50–1000 *m/z*. Peaks detected in CE-TOFMS analysis were extracted using automatic integration software (MasterHands ver. 2.17.1.11 developed at Keio University) in order to obtain peak information including *m/z*, migration time (MT), and peak area. The peak area was then converted to relative peak area by normalizing to the product of the internal standard area and the sample amount. The peak detection limit was determined based on a signal-noise ratio of 3. Putative metabolites were then assigned from HMT’s standard library and Known-Unknown peak library on the basis of *m/z* and MT. The tolerance was ±0.5 min in MT and ±10 ppm in *m/z*. Absolute quantification was performed in target metabolites. All the metabolite concentrations were calculated by normalizing the peak area of each metabolite with respect to the area of the internal standard and by using standard curves, which were obtained by single-point (100 μM) calibrations. Glycogen was quantitated using the a65620 Glycogen Assay Kit (Abcam) by recording the absorption spectra of the OxiRed probe after reaction of glucoamylase with lysed cell sample aliquots according to the manufacturer’s protocol.

## Supporting information

S1 Fig Simultaneous determination of ΔΨ and respiration rate when adding venturicidin after KCl. 2.5 x 10^9^ K^+^-depleted wild-type cells/ml grown in malate were incubated in depletion buffer to which 16.5 mM sodium malate was added at the indicated time point followed by 10 mM KCl and 10 μM venturicidin (Vent). ΔΨ (red) was determined as described in Methods overlayed with the O_2_ concentration trace (blue) showing respiration rates in mol O_2_/min/mol cytochrome *aa*_3_ recorded after the indicated additions.
(TIF)

S2 Fig Glycogen content in 2 x 10^9^ CyoB^-^/CcoN^-^ K^+^-depleted cells. After 20 min of incubation in depletion buffer supplemented with 16.5 mM sodium malate (left) or with 16.5 mM sodium malate and 10 mM KCl (right), cells were disrupted followed by reaction with glucoamylase and the OxiRed colorimetric probe that detects glucose released from glycogen hydrolysis (see Methods). The glucose background was recorded in sample lacking glucoamylase. An internal standard for quantitation was included by adding 1 μg of glycogen to sample aliquots.
(TIF)

S3 Fig Time courses of volume changes (A), oxygen consumption, and K^+^ uptake (B). 2 x 10^9^ K^+^ depleted wild-type cells/ml were incubated in the presence of 16.5 mM sodium malate followed by the addition of 10 mM KCl (red), or in the presence of 10 mM KCl followed by 16.5 mM sodium malate (blue). K^+^ uptake (green) is shown only for the experiment in which KCl was added after malate. Volume changes were recorded in parallel by monitoring the optical density at 630 nm.
(TIF)

S4 Fig **Heat generation rate determined by microcalorimetry (red) compared to respiration rate (blue).** Wild-type K^+^ depleted cells were respiring in 16.5 mM sodium malate in the presence or absence of 5 mM KCl. See Materials and Methods for experimental details. Bars represent the average ± standard error values of 5 independent determinations.
(TIF)

S5 Fig Simultaneous determination of ΔΨ and respiration rate adding cyanide after apparent oxygen depletion. 2.5 x 10^9^ K^+^-depleted wild-type cells/ml grown in malate were incubated in depletion buffer to which 16.5 mM sodium malate was added at the indicated time point followed by 10 mM KCl and 1 mM Na CN. ΔΨ (red) was determined as described in Methods overlayed with the O_2_ concentration trace (blue) showing respiration rates in mol O_2_/min/mol cytochrome *aa*_3_ recorded after the indicated additions.
(TIF)

S1 Table Relative expression of oxidative phosphorylation and K^+^ transport proteins. (DOCX)

S2 Table Most overexpressed and underexpressed proteins in wild-type *P*. *denitrificans* cells grown in glucose relative to malate. (DOCX)

S3 Table Most overexpressed and underexpressed proteins in CyoB^-^/CcoN^-^ cells grown in glucose compared to wild-type. (DOCX)

S4 Table Most overexpressed and underexpressed proteins in CyoB^-^/CcoN^-^ cells grown in malate compared to wild-type. (DOCX)

S5 Table Relative changes in the expression of all proteins detected as a function of substrate and *P*. *denitrificans* strain. (XLSX)

S6 Table Colony forming units (CFU) and optical density (OD) of wild-type cells in the presence and absence of NH4^+^ or KCl. (DOCX)
