# Supplementary figures and images for: Energy homeostasis is a conserved process: Evidence from Paracoccus denitrificans’ response to acute changes in energy demand
Source: PLoS One. 2021 Nov 8;16(11):e0259636. doi: 10.1371/journal.pone.0259636 (PMC8575270; doi:10.1371/journal.pone.0259636)

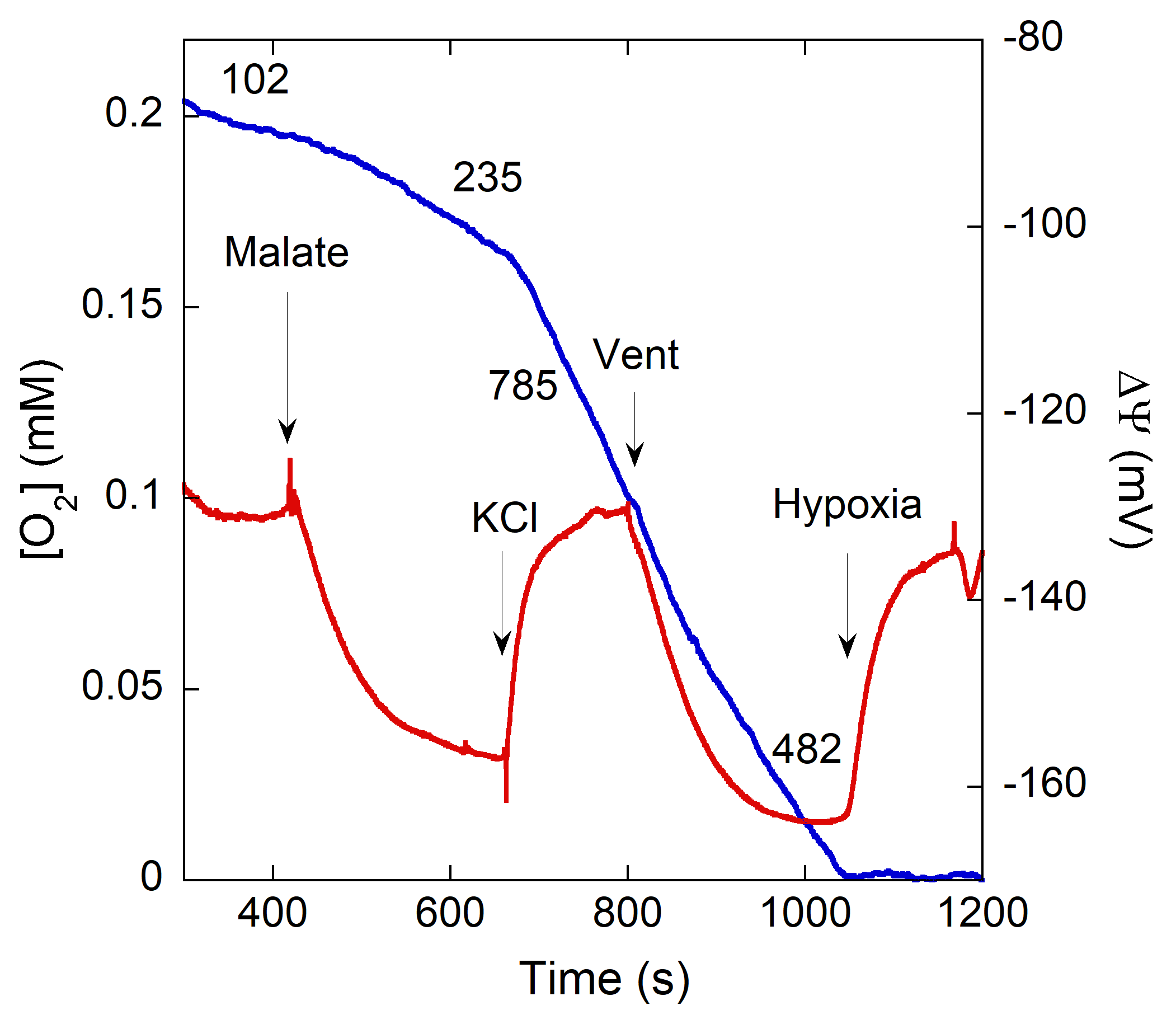

Supplement: S1 Fig — 2.5 x 10^9^ K+-depleted wild-type cells/ml grown in malate were incubated in depletion buffer to which 16.5 mM sodium malate was added at the indicated time point followed by 10 mM KCl and 10 μM venturicidin (Vent). ΔΨ (red) was determined as described in Methods overlayed with the O2 concentration trace (blue) showing respiration rates in mol O2/min/mol cytochrome aa3 recorded after the indicated additions. (TIF) [file pone.0259636.s001.tif]

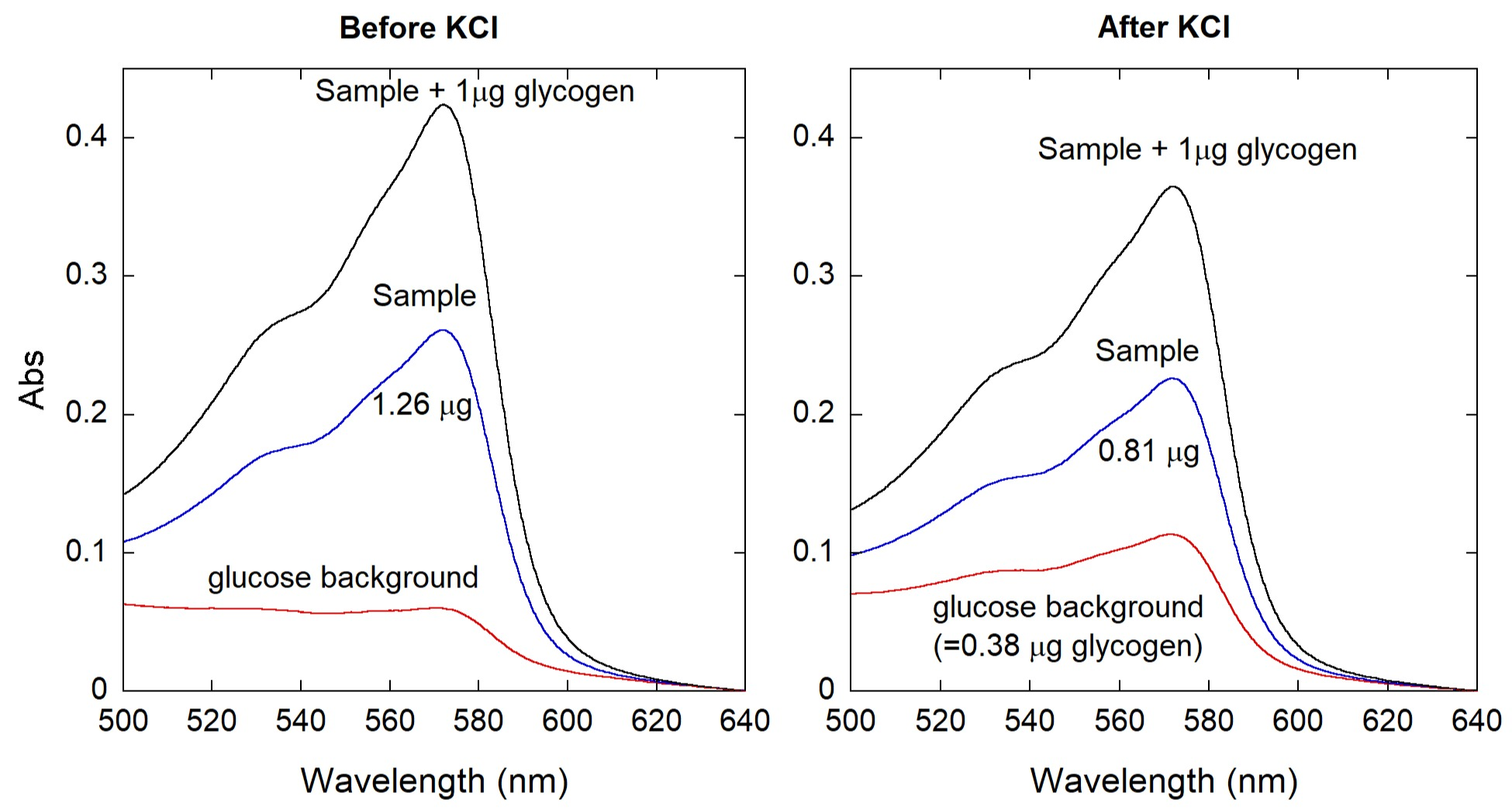

Supplement: S2 Fig — After 20 min of incubation in depletion buffer supplemented with 16.5 mM sodium malate (left) or with 16.5 mM sodium malate and 10 mM KCl (right), cells were disrupted followed by reaction with glucoamylase and the OxiRed colorimetric probe that detects glucose released from glycogen hydrolysis (see Methods). The glucose background was recorded in sample lacking glucoamylase. An internal standard for quantitation was included by adding 1 μg of glycogen to sample aliquots. (TIF) [file pone.0259636.s002.tif]

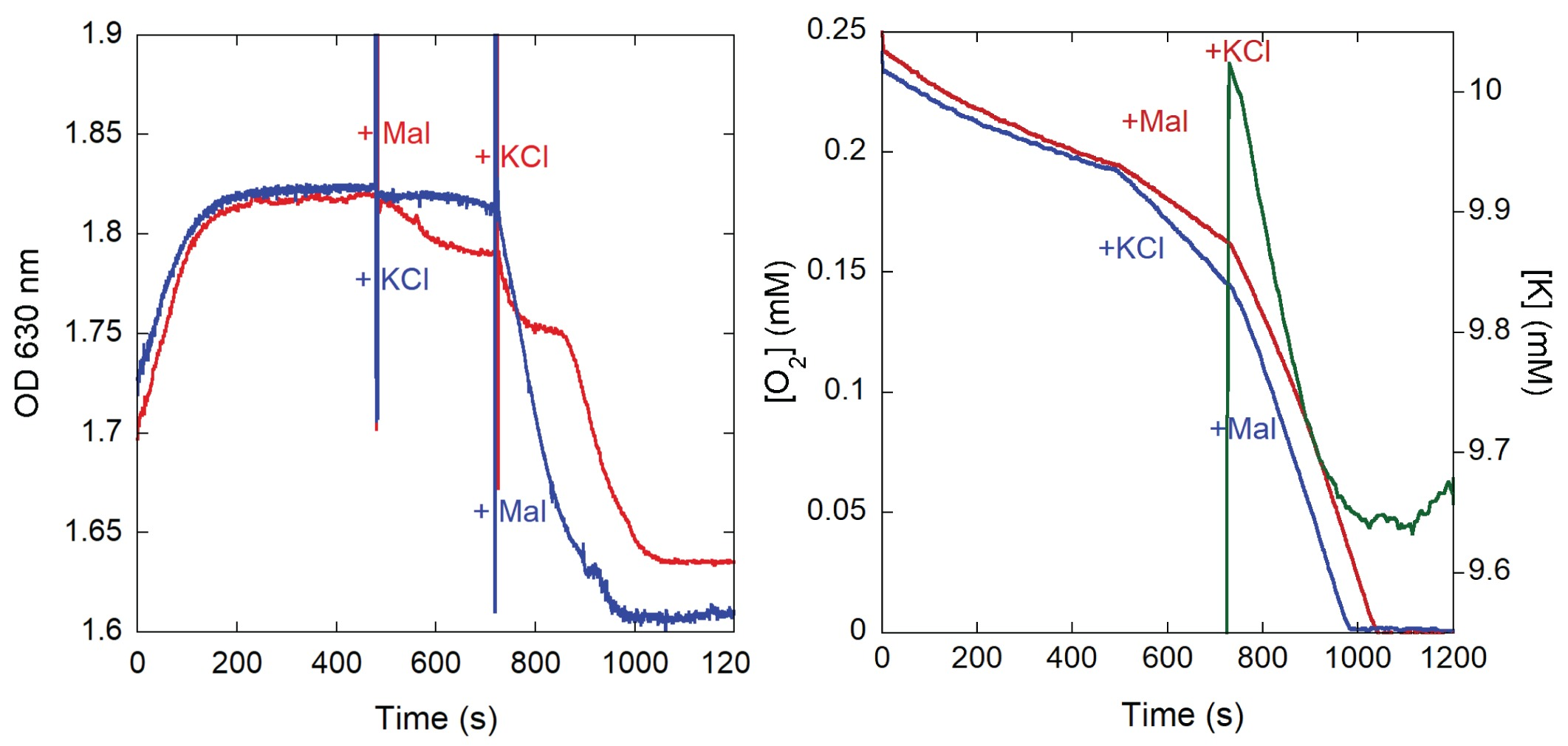

Supplement: S3 Fig — Time courses of volume changes (A), oxygen consumption, and K+ uptake (B). 2 x 10^9^ K+ depleted wild-type cells/ml were incubated in the presence of 16.5 mM sodium malate followed by the addition of 10 mM KCl (red), or in the presence of 10 mM KCl followed by 16.5 mM sodium malate (blue). K+ uptake (green) is shown only for the experiment in which KCl was added after malate. Volume changes were recorded in parallel by monitoring the optical density at 630 nm. (TIF) [file pone.0259636.s003.tif]

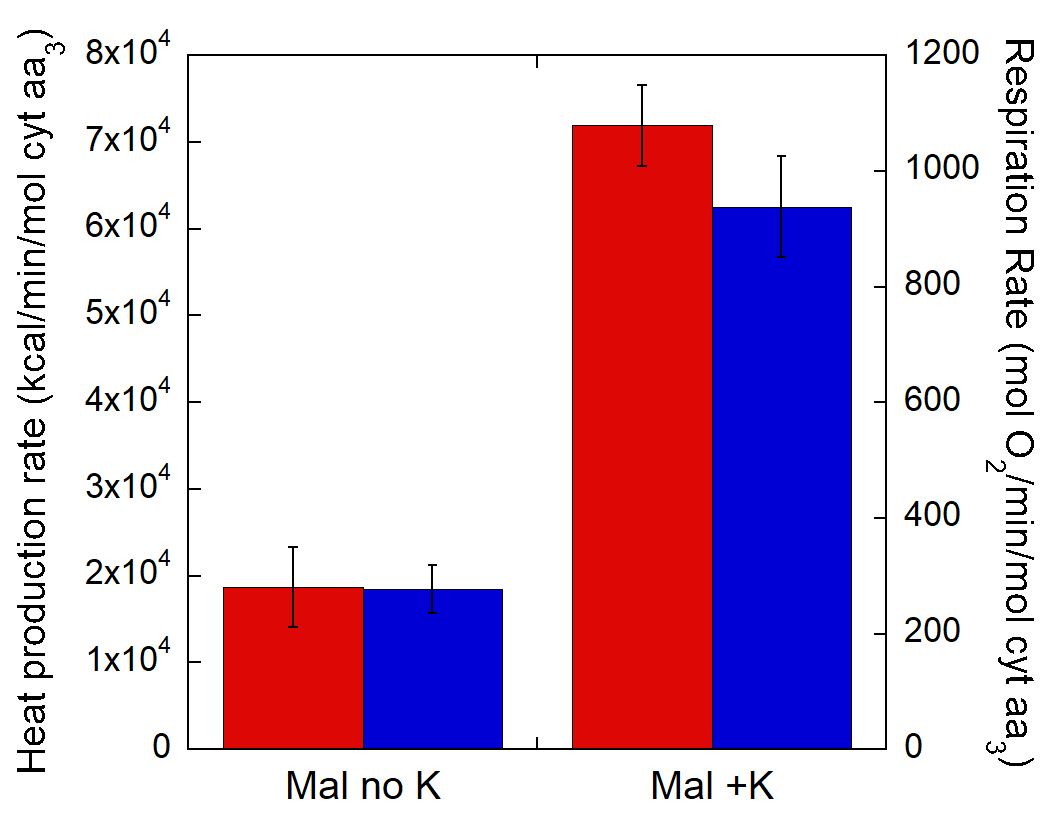

Supplement: S4 Fig — Heat generation rate determined by microcalorimetry (red) compared to respiration rate (blue). Wild-type K+ depleted cells were respiring in 16.5 mM sodium malate in the presence or absence of 5 mM KCl. See Materials and Methods for experimental details. Bars represent the average ± standard error values of 5 independent determinations. (TIF) [file pone.0259636.s004.tif]

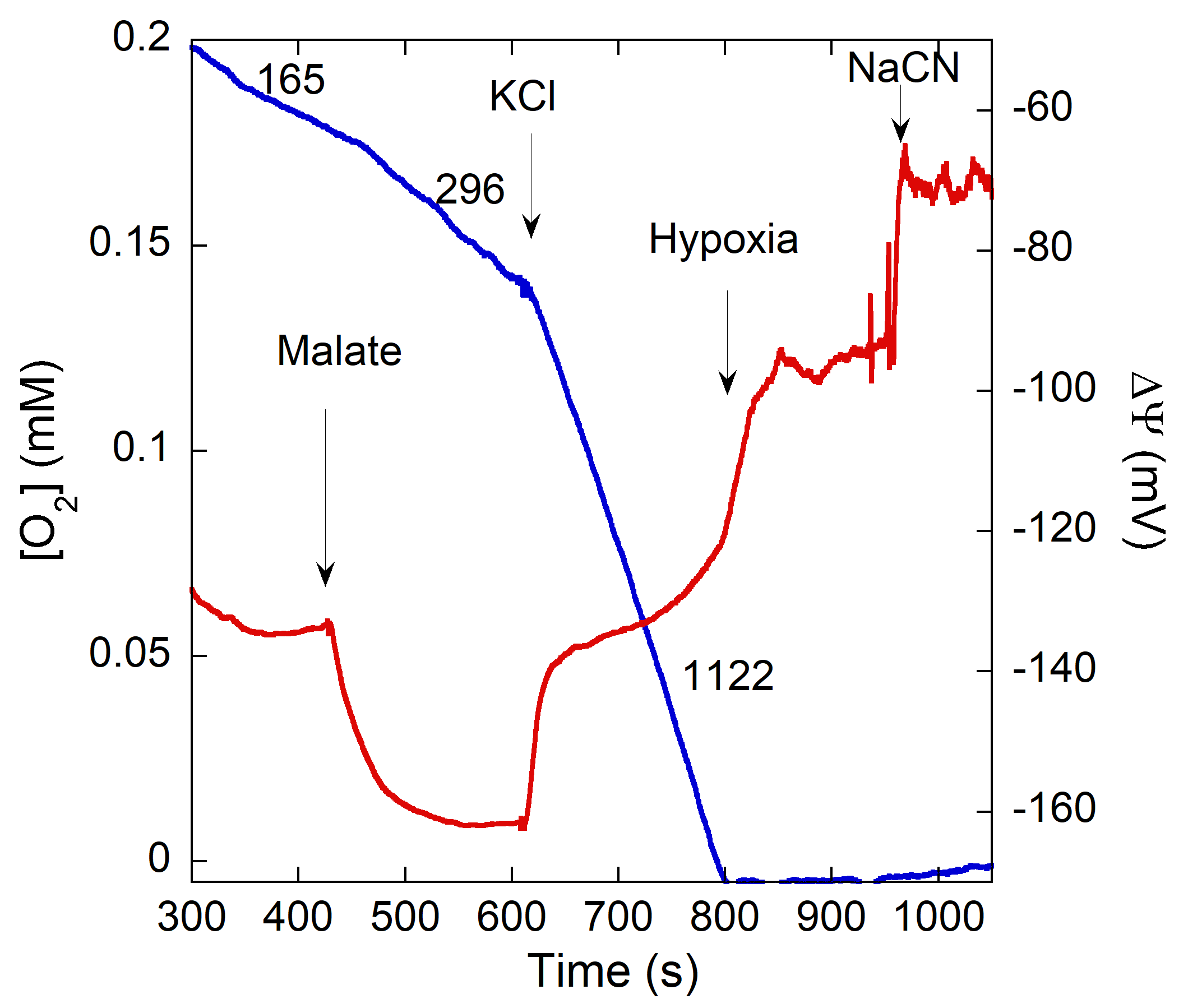

Supplement: S5 Fig — 2.5 x 10^9^ K+-depleted wild-type cells/ml grown in malate were incubated in depletion buffer to which 16.5 mM sodium malate was added at the indicated time point followed by 10 mM KCl and 1 mM Na CN. ΔΨ (red) was determined as described in Methods overlayed with the O2 concentration trace (blue) showing respiration rates in mol O2/min/mol cytochrome aa3 recorded after the indicated additions. (TIF) [file pone.0259636.s005.tif]
