# Supplementary material for: Energy homeostasis is a conserved process: Evidence from Paracoccus denitrificans’ response to acute changes in energy demand
Source: PLoS One. 2021 Nov 8;16(11):e0259636. doi: 10.1371/journal.pone.0259636 (PMC8575270; doi:10.1371/journal.pone.0259636)
Supplement: S1 Table — (DOCX) [file pone.0259636.s006.docx]

**S1 Table. Relative expression of oxidative phosphorylation and K^+^ transport proteins^a^**

| **Protein** | **WTGluc/**  **WTMal** | **dKOGluc/**  **dKOMal** | **dKOGluc/**  **WTGluc** | **dKOMal/**  **WTMal** |
| --- | --- | --- | --- | --- |
| **Complex I:** |  |  |  |  |
| Subunit A | 0.95 | 0.65 | 0.79 | 1.16 |
| Subunit B | 1.08 | 1.09 | 0.98 | 1.01 |
| Subunit C | 1.1 | 1.20 | 1.29 | 1.14 |
| Subunit D | 0.9 | 0.9 | 1.06 | 0.95 |
| Subunit E | 1.42 | 1.14 | 1.03 | 1.31 |
| Subunit F | 0.79 | 0.90 | 1.09 | 0.90 |
| Subunit G | 0.78 | 0.88 | 0.98 | 0.86 |
| Subunit H | 0.62 | 0.98 | 1.03 | 0.73 |
| Subunit I | 1.02 | 0.98 | 0.87 | 0.89 |
| Subunit J | 0.58* | 0.96 | 1.08 | 0.62 |
| Subunit K | 0.54* | 0.69 | 0.85 | 0.62 |
| Subunit L | 0.65 | 1.04 | 1.25 | 0.82 |
| Subunit M | 0.68 | 0.92 | 1.15 | 0.77 |
| Subunit N | 0.62 | 0.93 | 1.02 | 0.84 |
| Subunit NDUFA12 | 1.12 | 0.84 | 0.85 | 0.95 |
| **Complex III:** |  |  |  |  |
| Iron-sulfur subunit | 0.70 | 1.08 | 1.38 | 0.83 |
| Cytochrome *c*_1_ | 0.69 | 0.89 | 1.27 | 1.01 |
| **Other *c*-type cytochromes:** |  |  |  |  |
| Cytochrome *c*550 | 0.95 | 1.38 | 0.6* | 0.43* |
| Cytochrome *c*552 | 1.15 | 1.09 | 1.28 | 1.29 |
| **Complex IV:** |  |  |  |  |
| Subunit I | 0.78 | 1.06 | 1.06 | 0.9 |
| Subunit II | 0.61 | 1.09 | 1.47* | 0.74 |
| Subunit III | 0.66 | 0.71 | 0.85 | 0.78 |
| Subunit IV | 0.67 | 0.93 | 1.09 | 0.77 |
| **Alternative terminal oxidases:** |  |  |  |  |
| Ubiquinol oxidase subunit II | 0.13* | 0.67 | 1.58 | 0.3* |
| Cytochrome *cbb*_3_-type subunit II | 0.64 | 0.94 | 0.30* | 0.06* |
| Cytochrome *cbb*_3_-type subunit III | 0.56 | 0.43 | 0.03* | 0.04* |
| **Complex V:** |  |  |  |  |
| ATP synthase subunit alpha | 0.99 | 0.98 | 0.91 | 0.82 |
| ATP synthase subunit beta | 0.89 | 0.97 | 0.83 | 0.78 |
| ATP synthase subunit delta | 1.18 | 1.08 | 0.92 | 0.93 |
| ATP synthase subunit epsilon | 0.91 | 0.87 | 0.78 | 0.82 |
| ATP synthase subunit gamma | 1.05 | 1.06 | 0.96 | 0.85 |
| ATP synthase subunit a | 0.93 | 0.94 | 0.96 | 0.75 |
| ATP synthase subunit b | 0.92 | 0.97 | 0.98 | 0.87 |
| **K^+^ transport:** |  |  |  |  |
| Trk K^+^ transporter TrkA subunit | 1.08 | 0.96 | 1.34 | 1.52 |
| Trk K^+^ transporter TrkH subunit | 0.84 | 0.90 | 1.89* | 1.67 |
| K^+^/H^+^ antiporter PhaG subunit | 1.19 | 1.02 | 1.13 | 1.12 |

^a^Median ratios of all peptides detected for a particular protein are shown for each pairwise comparison. WTGluc = wild-type cells grown in glucose; WTMal = wild-type cells grown in malate; dKOGluc = CyoB^-^/CcoN^-^ cells grown in glucose; dKOMal = CyoB^-^/CcoN^-^ cells grown in malate.

*Values found to be significantly different (p<0.05; ANOVA).
