# Supplementary material for: Energy homeostasis is a conserved process: Evidence from Paracoccus denitrificans’ response to acute changes in energy demand
Source: PLoS One. 2021 Nov 8;16(11):e0259636. doi: 10.1371/journal.pone.0259636 (PMC8575270; doi:10.1371/journal.pone.0259636)
Supplement: S2 Table — (DOCX) [file pone.0259636.s007.docx]

**S2 Table. Most overexpressed and underexpressed proteins in wild-type *P. denitrificans* cells grown in glucose relative to malate^a^**

| **Protein** | **WTGluc/WTMal** |
| --- | --- |
| sn-glycerol-3-phosphate ABC transporter ATP-binding protein UgpC | 59.36 |
| quinohemoprotein amine dehydrogenase subunit beta | 42.90 |
| quinohemoprotein amine dehydrogenase maturation protein | 30.85 |
| ABC transporter ATP-binding protein | 28.88 |
| aldehyde dehydrogenase family protein | 27.58 |
| phosphogluconate dehydratase | 25.36 |
| ABC transporter substrate-binding protein | 20.65 |
| tripartite tricarboxylate transporter substrate binding protein | 20.40 |
| tripartite tricarboxylate transporter permease | 19.91 |
| 6-phosphogluconolactonase | 18.88 |
| Gfo/Idh/MocA family oxidoreductase | 18.56 |
| tripartite tricarboxylate transporter substrate binding protein | 18.52 |
| gluconokinase | 18.24 |
| Twin-arginine translocation pathway signal | 16.49 |
| trehalose utilization protein ThuA | 16.16 |
| tripartite tricarboxylate transporter substrate binding protein | 14.26 |
| Gfo/Idh/MocA family oxidoreductase | 14.11 |
| AMP-binding protein | 13.46 |
| quinohemoprotein amine dehydrogenase subunit alpha | 11.47 |
| GMC family oxidoreductase | 11.02 |
| NAD(P)-binding protein | 0.26 |
| LysR family transcriptional regulator | 0.25 |
| queuosine precursor transporter | 0.24 |
| LLM class flavin-dependent oxidoreductase | 0.24 |
| P4 family phage/plasmid primase | 0.23 |
| sensor histidine kinase | 0.23 |
| 50S ribosomal protein L36 | 0.23 |
| carbohydrate ABC transporter permease | 0.20 |
| acyl-CoA/acyl-ACP dehydrogenase | 0.19 |
| phosphoenolpyruvate carboxykinase | 0.18 |
| peroxiredoxin | 0.17 |
| malate synthase A | 0.15 |
| GNAT family N-acetyltransferase | 0.14 |
| ubiquinol oxidase subunit II | 0.13 |
| FAD-dependent oxidoreductase | 0.12 |
| ABC transporter ATP-binding protein | 0.11 |
| putative hydantoin racemase protein | 0.09 |
| DNA methylase N-4 | 0.08 |
| DUF521 domain-containing protein | 0.05 |
| dicarboxylate/amino acid:cation symporter | 0.02 |

^a^The 20 most overexpressed and underexpressed proteins in wild-type cells grown in glucose (WTGluc) relative to those grown in malate (WTMal) are shown. Ratios corresponds to the median of all peptide ratios for a particular protein.
