# Supplementary material for: Energy homeostasis is a conserved process: Evidence from Paracoccus denitrificans’ response to acute changes in energy demand
Source: PLoS One. 2021 Nov 8;16(11):e0259636. doi: 10.1371/journal.pone.0259636 (PMC8575270; doi:10.1371/journal.pone.0259636)
Supplement: S3 Table — (DOCX) [file pone.0259636.s008.docx]

**S3 Table. Most overexpressed and underexpressed proteins in CyoB^-^/CcoN^-^ cells grown in glucose compared to wild-type^a^**

| **Protein** | **dKOGluc/WTGluc** |
| --- | --- |
| FMN reductase | 3.21 |
| Amidohydrolase | 2.84 |
| ABC transporter permease | 2.53 |
| serine hydrolase | 2.34 |
| molybdate ABC transporter substrate-binding protein | 2.34 |
| PaaI family thioesterase | 2.29 |
| SoxS | 2.27 |
| translation initiation factor IF-3 | 2.24 |
| sulfur dehydrogenase SoxD | 2.21 |
| NAD(P)H-dependent oxidoreductase | 2.19 |
| DUF3775 domain-containing protein | 2.10 |
| 6,7-dimethyl-8-ribityllumazine synthase | 2.09 |
| SDR family oxidoreductase | 2.02 |
| Rrf2 family transcriptional regulator | 2.00 |
| metal-dependent hydrolase | 1.97 |
| Bax inhibitor-1/YccA family protein | 1.97 |
| MarR family transcriptional regulator | 1.97 |
| Cytochrome-c peroxidase | 1.96 |
| glycerol-3-phosphate 1-O-acyltransferase PlsY | 1.95 |
| DUF2200 domain containing protein | 1.92 |
| ABC transporter substrate-binding protein | 0.28 |
| peptidase S15 | 0.28 |
| carbohydrate ABC transporter substrate-binding protein | 0.26 |
| DUF1153 domain-containing protein | 0.25 |
| ABC transporter ATP-binding protein | 0.24 |
| CcoQ/FixQ family Cbb3-type cytochrome c oxidase assembly chaperone | 0.24 |
| cupin domain-containing protein | 0.23 |
| GNAT family N-acetyltransferase | 0.23 |
| PepSY domain-containing protein | 0.22 |
| Clp protease ClpP | 0.21 |
| lactate utilization protein | 0.21 |
| ABC transporter ATP-binding protein | 0.21 |
| glutathione S-transferase family protein | 0.19 |
| chemotaxis protein MotB | 0.18 |
| nuclear export factor GLE1 | 0.14 |
| malate synthase A | 0.12 |
| NAD(P)-binding protein | 0.11 |
| ABC transporter ATP-binding protein | 0.07 |
| N-formylglutamate amidohydrolase | 0.06 |
| cytochrome-c oxidase, cbb3-type subunit III | 0.06 |

^a^The 20 most overexpressed and underexpressed proteins in CyoB^-^/CcoN^-^ cells grown in glucose (dKOGluc) relative to wild-type cells (WTGluc) are shown. Ratios corresponds to the median of all peptide ratios for a particular protein.
