# Supplementary material for: Energy homeostasis is a conserved process: Evidence from Paracoccus denitrificans’ response to acute changes in energy demand
Source: PLoS One. 2021 Nov 8;16(11):e0259636. doi: 10.1371/journal.pone.0259636 (PMC8575270; doi:10.1371/journal.pone.0259636)
Supplement: S4 Table — (DOCX) [file pone.0259636.s009.docx]

**S4 Table. Most overexpressed and underexpressed proteins in CyoB^-^/CcoN^-^ cells grown in malate compared to wild-type^a^**

| **Protein** | **dKOMal/WTMal** |
| --- | --- |
| Exopolysaccharide synthesis, ExoD | 19.39 |
| PaaI family thioesterase | 12.18 |
| serine hydrolase | 8.30 |
| Bax inhibitor-1/YccA family protein | 7.96 |
| GNAT family N-acetyltransferase | 7.19 |
| 2-hydroxychromene-2-carboxylate isomerase | 6.58 |
| transcriptional regulator, MarR family | 5.03 |
| YggT family protein | 4.57 |
| helix-turn-helix transcriptional regulator | 4.49 |
| putative membrane protein YgcG | 4.38 |
| HD domain-containing protein | 4.31 |
| 5'-methylthioadenosine/S-adenosylhomocysteine nucleosidase | 4.25 |
| orotate phosphoribosyltransferase | 4.15 |
| polysaccharide deacetylase | 4.09 |
| Lrp/AsnC family transcriptional regulator | 3.95 |
| Lrp/AsnC family transcriptional regulator | 3.81 |
| topoisomerase-primase domain containing protein | 3.71 |
| NADPH-dependent F420 reductase | 3.69 |
| winged helix-turn-helix transcriptional regulator | 3.65 |
| hydroxyisourate hydrolase | 3.61 |
| DUF4326 domain-containing protein | 0.23 |
| site-specific integrase | 0.23 |
| glutathione S-transferase family protein | 0.22 |
| APH domain-containing protein | 0.20 |
| PepSY domain-containing protein | 0.19 |
| FAD-dependent oxidoreductase | 0.19 |
| DUF1491 family protein | 0.18 |
| Allergen V5/Tpx-1 family protein | 0.18 |
| carbohydrate ABC transporter permease | 0.11 |
| nuclear export factor GLE1 | 0.10 |
| GNAT family N-acetyltransferase | 0.10 |
| NAD(P)-binding protein | 0.09 |
| putative hydantoin racemase protein | 0.07 |
| DNA methylase N-4 | 0.07 |
| malate synthase A | 0.07 |
| cytochrome-c oxidase, cbb3-type subunit II | 0.06 |
| CcoQ/FixQ family Cbb3-type cytochrome c oxidase assembly chaperone | 0.06 |
| N-formylglutamate amidohydrolase | 0.04 |
| ABC transporter ATP-binding protein | 0.04 |
| cytochrome-c oxidase, cbb3-type subunit III | 0.03 |

^a^The 20 most overexpressed and underexpressed proteins in CyoB^-^/CcoN^-^ cells grown in malate (dKOMal) relative to wild-type cells (WTMal) are shown. Ratios corresponds to the median of all peptide ratios for a particular protein.
