# Supplementary material for: Energy homeostasis is a conserved process: Evidence from Paracoccus denitrificans’ response to acute changes in energy demand
Source: PLoS One. 2021 Nov 8;16(11):e0259636. doi: 10.1371/journal.pone.0259636 (PMC8575270; doi:10.1371/journal.pone.0259636)
Supplement: S6 Table — (DOCX) [file pone.0259636.s011.docx]

**S6 Table. Colony forming units (CFU) and optical density (OD) of wild-type cells in the presence and absence of NH_4_^+^ or KCl^a^**

| **Time (h)** | **Growth medium** | | **No NH_4_^+^**  **+ 10 mM KCl** | | **No NH_4_^+^** | |
| --- | --- | --- | --- | --- | --- | --- |
|  | **CFU** | **OD** | **CFU** | **OD** | **CFU** | **OD** |
| 0 | 9.2 x 10^7^ | 0.104 | 8.1 x 10^7^ | 0.102 | 7.3 x 10^7^ | 0.107 |
| 1 | 10.4 x 10^7^ | 0.128 | 9 x 10^7^ | 0.12 | 7.5 x 10^7^ | 0.114 |
| 3.1 | 16.4 x 10^7^ | 0.284 | 6.5 x 10^7^ | 0.153 | 7.2 x 10^7^ | 0.109 |

^a^Wild-type cells adapted to growth in glucose were thawed, washed, and incubated for the indicated time on 2-3 agar plates (for CFU counting) or in a shake flask (for OD measurements) or containing growth medium, depletion buffer without NH_4_^+^ supplemented with 10 mM KCl, or depletion buffer without NH_4_^+^ and without KCl.
